# PolyGraph – Flexible, Biocompatible & Electrically Optimized Graphene‐Polymer Composites for Next‐Generation Neural Interfaces

**DOI:** 10.1002/adhm.202505076

**Published:** 2026-05-12

**Authors:** Jack Maughan, Ian Woods, Cian O'Connor, Pablo Quintana‐Sarti, Eoin Caffrey, Jose M. Munuera, Tian Carey, Adrian Dervan, Alejandro López Valdés, Omar Mamad, Maeve A. Caldwell, Fergal J. O'Brien, Jonathan N. Coleman

**Affiliations:** ^1^ School of Physics Trinity College Dublin (TCD) Dublin Ireland; ^2^ Tissue Engineering Research Group (TERG) Royal College of Surgeons in Ireland (RCSI) Dublin Ireland; ^3^ Centre for Research on Adaptive Nanostructures and Nanodevices (CRANN) Trinity College Dublin (TCD) Dublin Ireland; ^4^ Advanced Materials and BioEngineering Research (AMBER) Centre RCSI and TCD Dublin Ireland; ^5^ Department of Physiology & Medical Physics Royal College of Surgeons in Ireland (RCSI) Dublin Ireland; ^6^ FutureNeuro Research Ireland Centre for Translational Brain Science Royal College of Surgeons in Ireland (RCSI) Dublin Ireland; ^7^ Instituto de Ciencia y Tecnología del Carbono INCAR‐CSIC Oviedo Spain; ^8^ Department of Electronic and Electrical Engineering School of Engineering Trinity College Dublin (TCD) Dublin Ireland; ^9^ Trinity Centre for Biomedical Engineering (TCBE) Trinity College Dublin (TCD) Dublin Ireland; ^10^ Trinity College Institute of Neuroscience (TCIN) Trinity College Dublin (TCD) Dublin Ireland; ^11^ Global Brain Health Institute (GBHI) Trinity College Dublin (TCD) Dublin Ireland; ^12^ Discipline of Physiology, School of Medicine Trinity College Dublin (TCD) Dublin Ireland

**Keywords:** biocompatible nanomaterials, electroactive biomaterials, flexible neural interfaces, graphene‐polymer composites, neural stimulation, soft bioelectronics

## Abstract

Neural interfacing materials must deliver exceptional electrochemical performance, while integrating safely with the central nervous system. In this study we develop PolyGraph, a flexible, conductive, and biocompatible graphene‐polycaprolactone (PCL) nanocomposite designed to strike this balance, which enables fabrication of conformable multichannel microelectrode arrays. Optimized liquid‐phase exfoliation produces conductive, biocompatible PVP‐stabilized graphene nanosheets, which are incorporated into PCL to form flexible, processable composites – PolyGraph. This material demonstrates bio‐ and immuno‐compatibility with sensitive primary and iPSC‐derived neuronal and glial cells. PolyGraph achieves low impedance (∼1.6 Ω cm^2^ @ 1 kHz) and high charge injection capacity (11.7 mC/cm^2^ for a 100 ms pulse), enhanced by NaOH surface roughening and AuPd coating. Leveraging their processability, PolyGraph composites are fabricated into flexible, individually isolated microneedle electrode arrays with biomimetic soft hyaluronic acid backings. These arrays demonstrate bidirectional neural interfacing capabilities, enabling both the delivery of controlled stimulation pulses in physiological buffer and high‐resolution neuronal recording in murine brain slices, with machine learning‐based event classification. Together, these advances establish PolyGraph as an optimal material platform for next‐generation brain‐computer interfaces and soft bioelectronic devices.

## Graphical abstract

PolyGraph, a flexible graphene‐polycaprolactone nanocomposite, unites conductivity, biocompatibility, and processability for next‐generation neural interfaces. Fabricated into microneedle arrays with ultra‐flexible backings, PolyGraph enables bidirectional neuronal recording and stimulation in brain tissue, advancing brain‐computer interface (BCI) and soft bioelectronic applications.

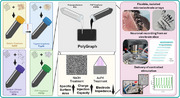

## 1 Introduction

Over the past half‐century, advances in our understanding of the central nervous system (CNS) have paralleled a revolution in neural interfacing technologies – devices that are capable of real‐time observation and modulation of neural activity, which have begun to revolutionize the treatment of epilepsy, Parkinson's disease and paralysis [1, 2, 3, 4, 5, 6, 7, 8, 9, 10]. Furthermore, advances in brain‐computer interface (BCI) technology even offer the prospect of seamless human‐machine communication, as resolution and interpretation improve [2, 11, 12]. Although remarkable progress has been made in microneedle arrays, neural threads, and mesh electronics, these neural interfacing technologies remain far from joining the clinical mainstays, electroencephalography (EEG) [13, 14] and functional magnetic resonance imaging (fMRI). This is despite their inability to directly modulate neural activity, alongside the low spatial resolution of EEG [15], and the high cost and limited portability of fMRI [16, 17], leading to significant demand for high‐resolution bidirectional neural interfaces. The gulf between technological development and clinical implementation underscores the challenges involved – devices must offer durable function and high resolution, without inducing scarring, infection, or device degradation [11]. These barriers do not arise from lack of innovation on device design, signal processing, or surgical techniques, but rather due to fundamental limitations of existing neural interfacing materials [4, 7, 18].

Novel electroconductive materials that combine biocompatibility, flexibility, and robust electrochemical performance are thus critical for advancing next‐generation neural interfaces. Conductive polymers have made substantial progress in addressing mechanical mismatch and improving electrochemical performance, emerging as a leading class of soft electrode materials [19, 20, 21, 22]. However, remaining challenges related to mechanical robustness, long‐term stability, and processing motivate exploration of complementary material systems, including conductive nanomaterials and nanocomposites [22, 23, 24, 25, 26, 27] In parallel, the incorporation of conductive polymers and nanomaterials within hydrogels has emerged as a rapidly advancing strategy, offering enhanced biological integration for neural interfacing applications [28, 29, 30].

2D nanomaterials, consisting of high‐aspect ratio, few‐atom thick sheets are particularly well‐positioned to address these challenges due to their exceptional electrical, mechanical, morphological, and processing properties [23, 24, 25, 26]. They offer a compelling alternative to metals and doped semiconductors, which often provoke fibrotic responses due to mechanical mismatch, hindering widespread long‐term clinical translation [31, 32, 33]. However, harnessing the full potential of 2D nanomaterials requires enhancing hydrophilicity and biocompatibility via surface functionalization [34, 35, 36]. Low molecular weight stabilizers (e.g. N‐Methyl‐2‐pyrrolidone, sodium cholate) yield excellent electrical performance [37, 38, 39] but poor biocompatibility, while biopolymers (e.g. gelatin, bovine serum albumin) improve biocompatibility, at the expense of electrical performance [40, 41, 42, 43, 44]. Emerging stabilizers such as pyrene derivatives [45, 46], polyethylene glycol, and polyvinylpyrrolidone (PVP) show promise in striking this balance, though performance remains highly application‐dependent [47, 48, 49, 50, 51].

Pairing 2D materials with biosafe polymers offers a promising route to achieving electrical performance, bioactivity, and desirable mechanical properties simultaneously [52, 53, 54]. Polycaprolactone (PCL), a biocompatible and processable thermoplastic [55], is one such polymer, enabling 3D printing, casting, and molding of nanocomposites [56, 57]. However, compared to both traditional and purely 2D systems [23, 58], fewer studies have explored the use of nanomaterial composites for neural interfacing electrodes, despite their potential to address many of the issues present in existing devices [19, 59]. In this work, we develop PolyGraph, a neural interface material with exceptional electrochemical performance, robust biocompatibility, and mechanical flexibility, based on PVP‐stabilized graphene embedded in PCL. While both stabilized graphene dispersions and PCL composites have been explored individually, their integration into an electrochemically optimized microneedle bidirectional neural interface remains unexplored.

Effective neural interfacing requires integration of novel materials into microelectrode [60, 61], microneedle [62, 63], or soft biohybrid [64, 65] architectures, which have dramatically improved both in vitro and clinical neural interfacing capabilities. The smaller addressable volume of these micro‐scale electrodes enables more precise control of external devices (e.g. prosthetics, communicators, computers), more accurate interpretation of neural signals, and a deeper understanding of CNS function [66, 67]. In this study, we fabricate a PolyGraph microneedle‐based electrode array with PDMS sheathing to reduce electrode size, and a flexible, biomimetic, immunomodulatory hyaluronic acid backing for conformability with neural tissues, decoupling the micromotion of the brain from the device [31, 68]. We demonstrate its capacity for bidirectional neural interfacing, recording neuronal activity from murine brain slices, and delivering stimulation pulses in physiological conditions.

In summary, this study introduces PolyGraph, a soft, flexible, biocompatible, and electrochemically optimized graphene‐polymer composite developed to address the trade‐offs of conductivity, biocompatibility, and mechanical mismatch in neural interfaces. We first identified PVP‐stabilized graphene incorporated into PCL as an optimal formulation, creating versatile, highly conductive, and biocompatible nanocomposites, which were subsequently engineered for enhanced electrochemical performance via NaOH surface roughening and AuPd coating. Leveraging the processability of these composites, we fabricated a multi‐channel microneedle‐based electrode array with a soft, biocompatible hyaluronic acid backing for conformability with neural tissues. Finally, we demonstrated the bidirectional functionality of PolyGraph through extracellular neuronal recording in ex vivo murine brain slices, coupled with machine learning‐based classification of neural events to enable real‐time interpretation, and stimulation in physiological medium.

By adapting established biomaterials to a promising microneedle fabrication method, and bolstered by extensive material and biological characterization, PolyGraph addresses key limitations of existing microelectrode designs, uniting softness, biocompatibility, and versatile, scalable fabrication. This establishes the potential of PolyGraph for next‐generation brain‐computer interfaces and soft bioelectronics, such as neural meshes, threads, microelectrode arrays, and deep brain stimulation devices [69, 70, 71, 72, 73, 74, 75, 76, 77].

## 2 Results and Discussion

### 2.1 Optimization of Graphene Formulation from Range of Candidate Exfoliations

#### 2.1.1 Physical Characteristics

For bioelectronics applications, there is a strong clinical need for soft materials with high conductivity and excellent biocompatibility. We first aimed to overcome the trade‐off between these two properties by identifying an optimal graphene stabilizer for use in the CNS. Complicating this optimization is the fact that many biocompatible stabilizers used to reduce nanomaterial cytotoxicity also significantly hamper their conductivity, due to steric hindrance and the disruption of nanosheet junctions [78, 79]. To address this, we evaluated four stabilizers for graphene liquid‐phase exfoliation (LPE) (Figure S1A).

**FIGURE 1:**
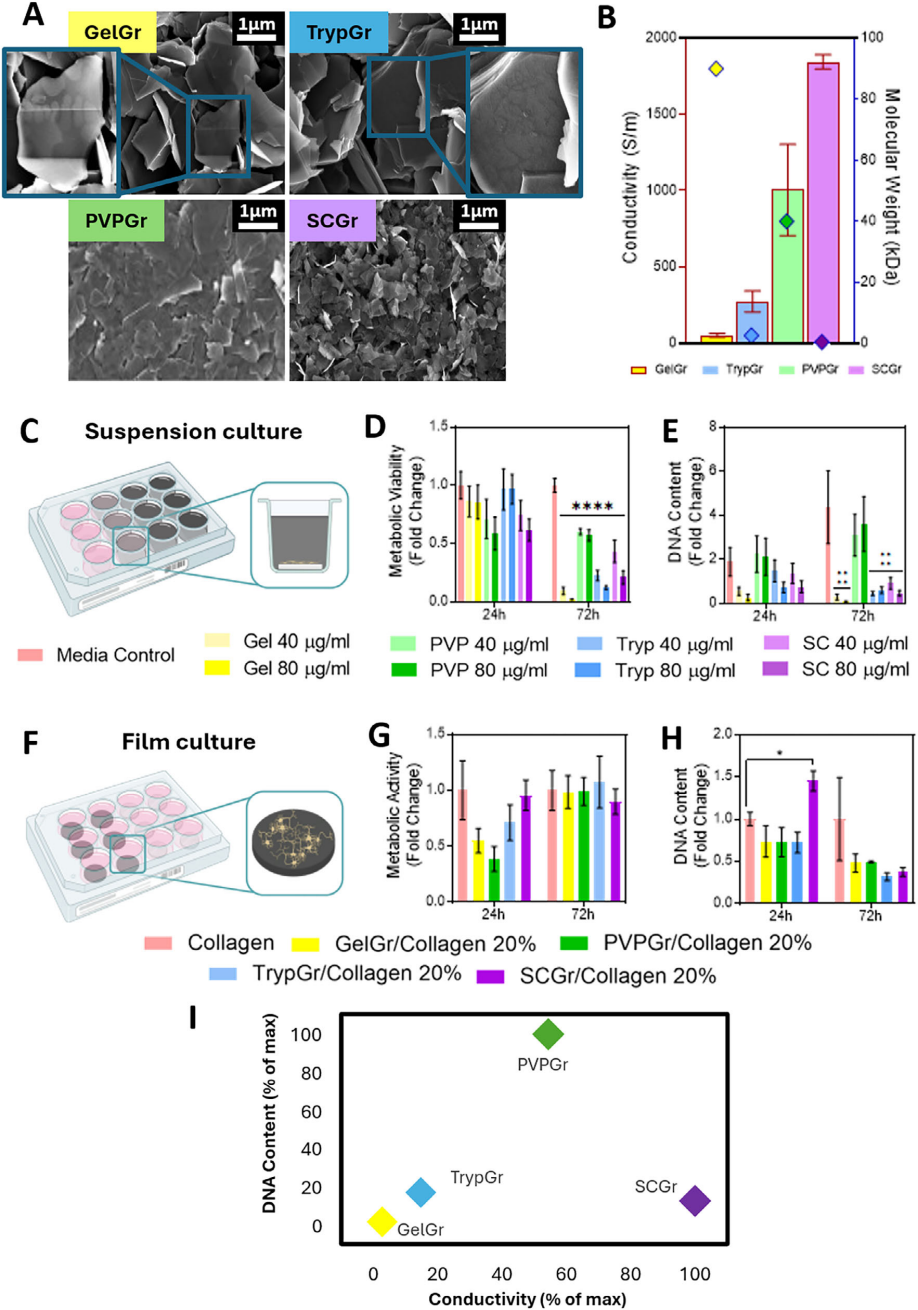
Physical characterization of graphene candidates: (A) SEM imaging of GelGr, TrypGr, PVPGr and SCGr formulations. Inset highlights disruption of gelatin layer by trypsin treatment. Scale bars 1 µm. (B) Conductivity of graphene films (bars), with molecular weights of stabilizers indicated by diamonds. (C) Schematic of suspension biocompatibility assays. (D) Metabolic activity and (E) DNA content of NSC‐34 cells grown in suspension with 40 or 80 µg/mL of each graphene formulation. (F) Schematic of collagen film biocompatibility assays. (G) Metabolic activity and (H) DNA content of NSC‐34 cells grown on collagen films containing 20 wt.% graphene. (I) Plot of cellular survival versus conductivity, expressed as percentage of maximum DNA content and conductivity for each graphene type. Significances: *p < 0.05, **p < 0.01, ***p < 0.001, ****p < 0.0001.

Optimization began with gelatin‐coated graphene (GelGr), which exhibited low conductivity (12 S/m), likely due to junction disruption by gelatin (Figure 1A), with composites fabricated using this graphene (Figure S2A) failing to reach the range required for low‐impedance, high‐resolution neural interfacing [80]. To probe the effect of stabilizer molecular weight, we digested the gelatin layer enzymatically using trypsin [81] (TrypGr). Conductivity measurements revealed a significant improvement (Figure 1B), supporting our hypothesis that thinner stabilizer coatings improved nanosheet junction quality and network conductivity. Thermogravimetric (TGA) analysis corroborated this, showing reduced stabiliser content in TrypGr (0.82%) compared to GelGr (4.64%)(Figure S3), while scanning electron microscopy (SEM), revealed disruption of the gelatin coating (inset, Figure 1A). Based on this evidence highlighting the importance stabilizer molecular weight to conductivity, we next tested two well‐established exfoliants with lower molecular weights than gelatin: sodium cholate (SC) [82] and polyvinylpyrrolidone (PVP) [48, 51]. Both yielded significantly higher conductivities and thinner, higher quality nanosheets than GelGr and TrypGr (Figure 1A,B). This is effect is posited to be due to two simultaneous and interacting factors: the effect of stabilizer molecular weight on junction quality and network conductivity, as evidenced by the difference between GelGr and TrypGr, and the effect of exfoliation efficiency of the stabiliser, which influences the size distribution, thickness, and stability of the nanosheets, and is determined by factors such as the surface energy and charge density [37, 83]. This effect can be seen in the differences between GelGr/TrypGr, PVPGr, and SCGr.

#### 2.1.2 Biological Characteristics

Having compared their physical characteristics, we next assessed candidate biocompatibility. To robustly examine the relative cytotoxicity of the nanoparticles, mouse motor neurons (NSC‐34s) were cultured with high‐concentration suspensions of each material (80 µg/mL) for 3 days (Figure 1C). This approach models an acute release of nanomaterial into the body – the highest risk scenario for cellular internalization, wrapping, and other damaging cell‐nanomaterial interactions [84, 85]. Cellular health and survival, assessed by metabolic activity (Figure 1D) and DNA content (Figure 1E), revealed significant cytotoxicity for all formulations, with the exception of PVPGr, which maintained healthy neuronal growth and metabolic activity. This striking result underscores the critical impact of stabilizer choice on nanomaterial biocompatibility. PVP, a commonly used biopolymer [47, 48], likely enhances biocompatibility due to steric shielding, protecting cells from direct contact with the sharp nanomaterial, and prevention of protein adsorption [86, 87]. This is consistent with findings for aromatic group‐based stabilizers such as bis‐pyrene [34, 45] and polyethylene glycol (PEG) [86, 88, 89].

Recognizing that immobilization of nanomaterials within a polymer matrix to form a nanocomposite can significantly enhance biocompatibility, we next assessed neuronal viability on collagen‐graphene composite films with each candidate graphene formulation (20 wt%, Figure 1F). In this case, the collagen matrix and reduced concentration of free graphene led to no significant reduction in cell population after 72 h (Figure 1G; Figure S1B), suggesting that the polymer matrix effectively mitigated cell‐nanomaterial interactions, reducing cytotoxic effects. The data thus far highlights PVP‐stabilized graphene (PVPGr) as the optimal candidate for further study, exhibiting a strong balance of biocompatibility with supra‐physiological conductivity (Figure 1I), positioning it as a promising material for use in the CNS.

### 2.2 PVP‐Stabilized Graphene (PVPGr) Characterization

**FIGURE 2:**
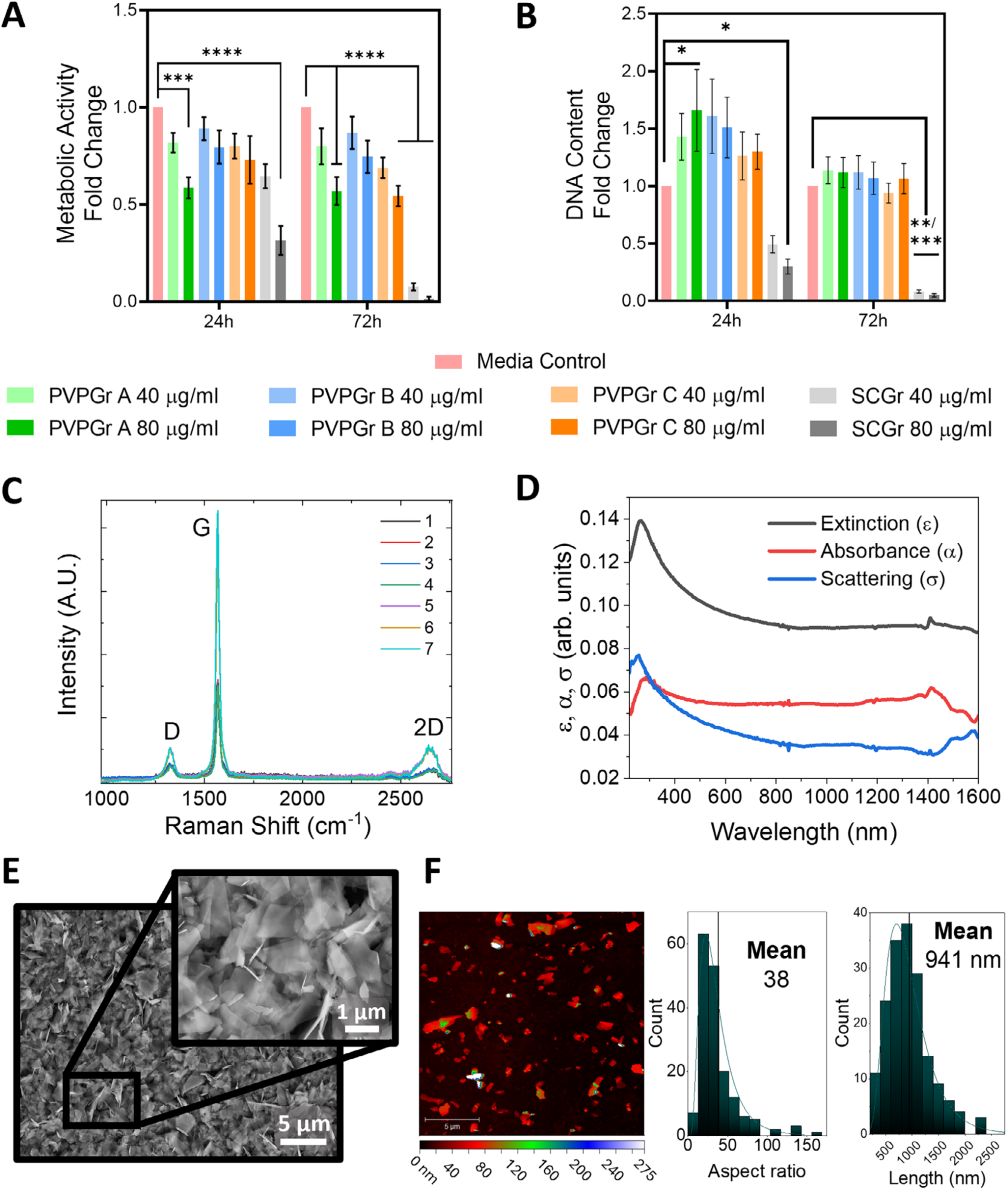
Physical & biological characterization of PVP‐stabilized graphene (PVPGr): (A, B) Metabolic activity (A) and DNA content (B) of NSC‐34 cells in suspension with PVPGr formulations, showing robust biocompatibility. (C) Raman spectra of PVPGr, with D, G, and 2D peaks characteristic of minimally‐defected graphene sheets. (D) UV–vis spectrum of PVPGr dispersion, with absorption and scattering features typical of graphene and nanomaterials. (E) SEM images of a PVPGr network, with inset showing thin, overlapping nanosheets. Main scale bar 5 µm, inset scale bar 1 µm. (F) AFM analysis of PVPGr, showing high aspect ratio and lateral size. Scale bar 5 µm. Significances: *p < 0.05, **p < 0.01, ***p < 0.001, ****p < 0.0001.

To confirm the robustness of the properties of PVP‐stabilized graphene (PVPGr) across a range of nanosheet morphologies and exfoliation parameters, we exposed mouse motor neurons to PVPGr formulations derived from distinct LPE protocols (Table 1). All formulations maintained largely stable neuronal metabolic activity (Figure 2A) and DNA content (Figure 2B), outperforming sodium cholate‐stabilized graphene (a commonly used stabilizing surfactant). This finding underscores the broad biocompatibility of PVPGr, and its relative independence from the precise exfoliation protocol.

To assess the basal plane quality and lateral size of PVPGr nanosheets, Raman spectroscopy was next performed (Figure 2C). Based on analysis of the spectra (Figure S4C), we determined that the nanosheets had largely undefected basal planes, and lateral sizes of 1.13 – 3.4 µm.

UV–vis spectroscopy showed the characteristic spectra of few‐layer graphene [90, 91], with a pronounced absorbance peak occurring at 284 nm (Figure 2D), alongside a significant degree of scattering, typical of nanomaterials [92]. SEM imaging revealed thin, evenly sized nanosheets (Figure 2E), while atomic force microscopy (AFM) analysis yielded a mean nanosheet length of 0.941 µm and aspect ratio of ∼38 (Figure 2F; Figure S5), aligning with Raman estimates. Collectively, these results confirm the production of high‐quality, biocompatible graphene sheets ideally suited for soft bioelectronics applications.

### 2.3 Manufacture and Optimization of PCL‐Graphene Composite (PolyGraph) Materials

Polymer‐free nanomaterial networks often suffer from fragility, as their cohesion arises due to weak van der Waals interactions, leaving them prone to delamination and degradation [93]. To overcome this, we developed a composite system harnessing the mechanical robustness and processability of polymers, while preserving the exceptional properties of graphene. The chosen polymer matrix needed to meet several criteria, including low molecular weight to minimize steric hindrance and maintain nanosheet conductivity, along with biological stability and biocompatibility. Polycaprolactone (PCL), a slow‐degrading biocompatible thermoplastic widely used in biomedical applications [55, 94, 95, 96], was selected for its versatile processability (3D printing, casting, and molding) [56]. Its relatively low modulus (∼350 MPa [97]), two orders of magnitude lower than gold [98] and three orders of magnitude lower than platinum or silicon [99, 100], is expected to enhance cellular adhesion and proliferation, while reducing stiffness‐induced foreign body response [101].

**FIGURE 3:**
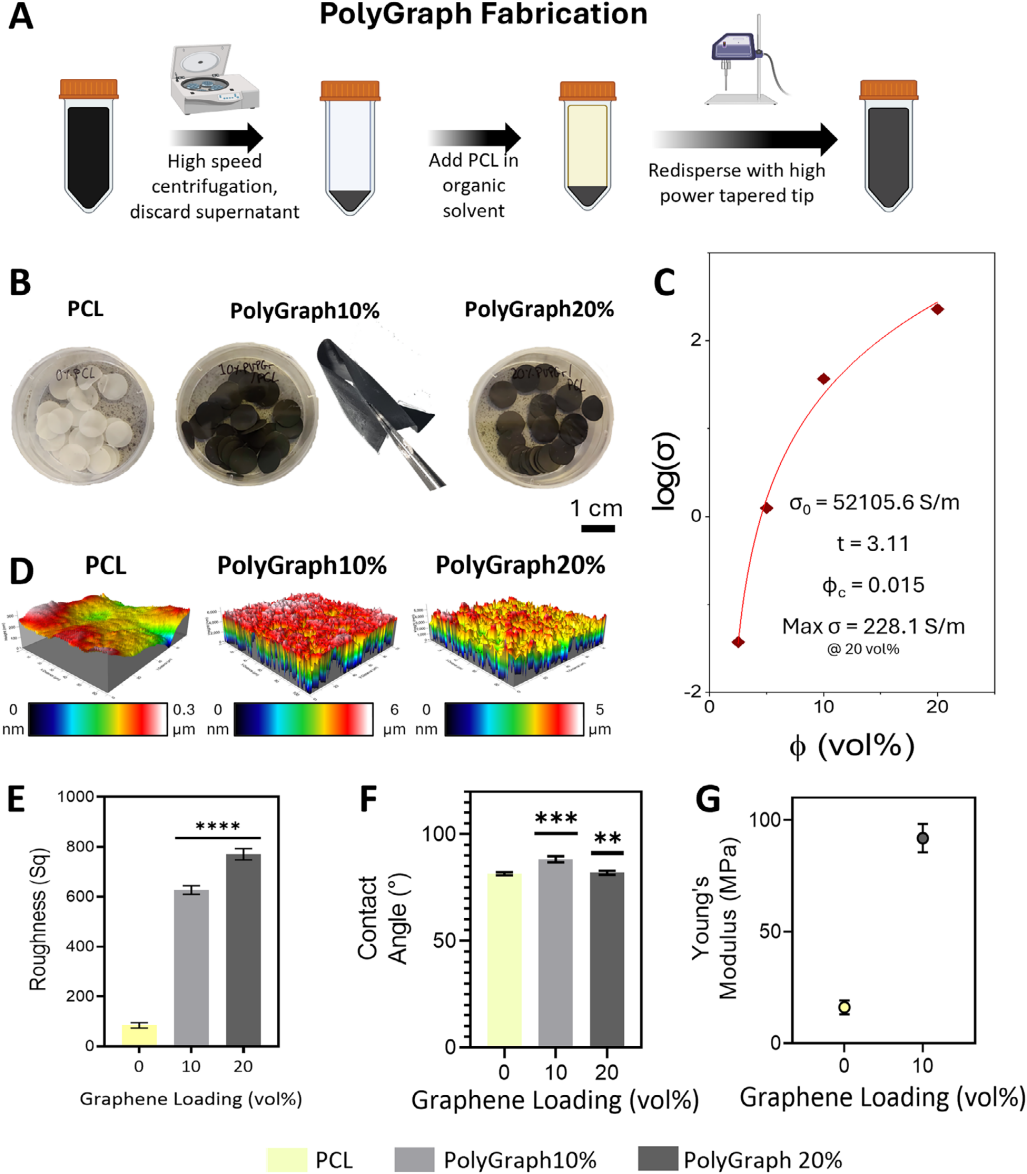
Physical characterization of PolyGraph composites: (A) Schematic of PolyGraph fabrication process. (B) Optical images of PCL and PolyGraph. (C) Percolation curve for PolyGraph conductivity with fitting parameters (percolation threshold Φ_c_, network conductivity σ_0_, critical exponent t). (D, E) White light interferometry data (D) for PCL and PolyGraph, indicating increased roughness with graphene loading (E). (F) Contact angle measurements for PolyGraph composites, indicating a slight but significant increase in hydrophobicity with graphene loading. (G) Young's modulus of PolyGraph, showing increased tensile strength with graphene loading. Significances: *p < 0.05, **p < 0.01, ***p < 0.001, ****p < 0.0001.

The optimized manufacturing process (Figure 3A) produced flexible PCL‐graphene (PolyGraph) composite films with minimal aggregation. To balance conductivity and mechanical properties, we assessed the percolation threshold in a graphene loading range of 0.125 to 20 vol% (Figure 3C). Percolation was reached at ∼1.45 vol%, with a critical exponent of 3.1, higher than the value of *t = 2* predicted by theory [102, 103] – this may be explained by the broadening of the distribution of both nanosheet and junction resistances due to variability in polymer coating and sheet quality (Figure S2) [102, 103, 104, 105]. Based on these results, 10 vol% (PolyGraph10%, ∼36 S/m) and 20 vol% (PolyGraph20%, ∼228 S/m) were chosen for further study, as they exhibited the best balance between electrical and mechanical properties, suitable for CNS applications.

Both formulations yielded homogeneous, flexible films (Figure 3B). Surface roughness increased with graphene loading (Figure 3D,E), disrupting the smooth polymer surface in a manner known to promote cellular adhesion [106, 107, 108] and enhance electrochemical performance [109, 110].

Contact angle measurements (Figure 3F) revealed a slight decrease in hydrophilicity for PolyGraph samples. Finally, TGA confirmed graphene loadings matched target fractions (Figure S4A,B). Mechanical testing showed an increase in Young's modulus with graphene addition (Figure 3G), accompanied by reduced ductility (Figure S6). This decrease is consistent with high filler loadings, where weak inter‐flake interactions begin to dominate, reducing composite strength and ductility, while also changing its viscoelastic properties [111]. This is corroborated by the significant embrittlement and handling difficulties observed for PolyGraph20%, which precluded reliable tensile testing. Collectively, these findings position PolyGraph as a flexible, mechanically robust, and electrically optimised platform for advanced soft bioelectronics.

### 2.4 Response of Neuronal and Glial Cells to PolyGraph Composites

#### 2.4.1 Neuronal Response

**FIGURE 4:**
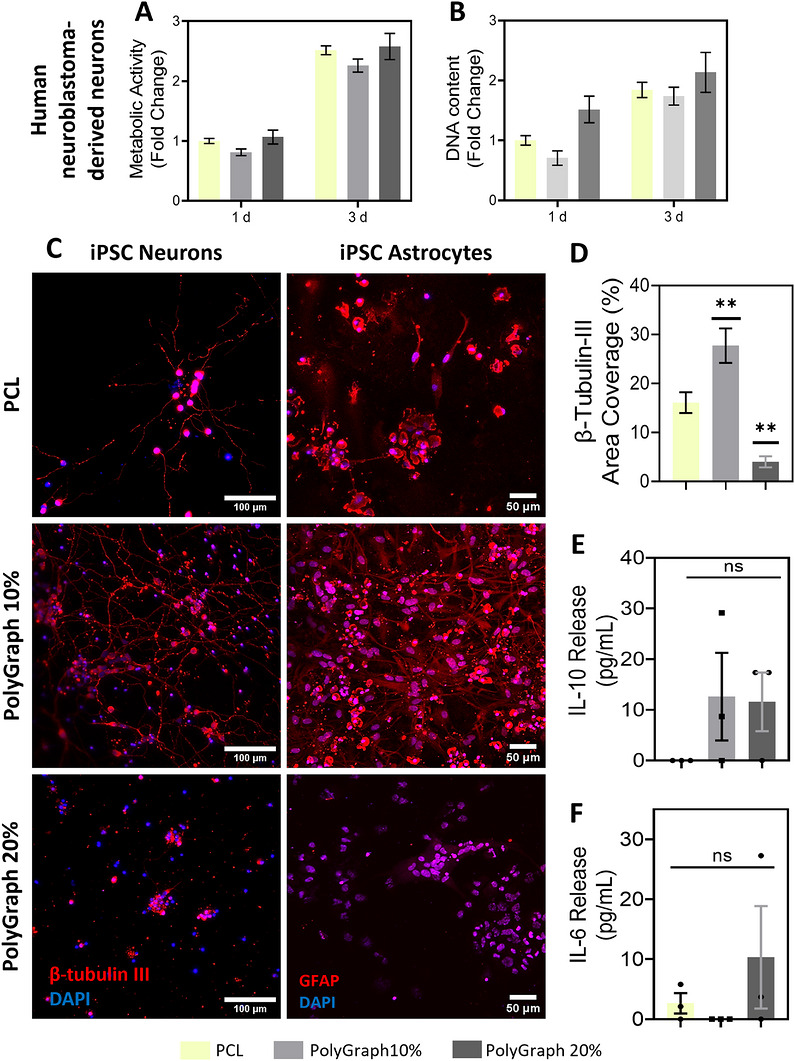
Biological & immunological characterization of PolyGraph: (A, B) Metabolic activity (A) and DNA content (B) of SH‐SY5Y neurons cultured on PolyGraph over 3 days. (C) Representative immunofluorescence images of iPSC‐derived neurons and astrocytes after 14 days on PolyGraph, demonstrating robust survival, healthy morphologies, and extensive neurite outgrowth across the surface of PolyGraph10%. Scale bars: neurons 100 µm, astrocytes 50 µm. (D) Quantification of β‐III tubulin coverage, indicating increased neuronal coverage on PolyGraph10%. (E, F) Cytokine release from iPSC‐derived astrocytes: E) anti‐inflammatory IL‐10, and F), pro‐inflammatory IL‐6. Significances: *p < 0.05, **p < 0.01, ***p < 0.001, ****p < 0.0001.

**FIGURE 5:**
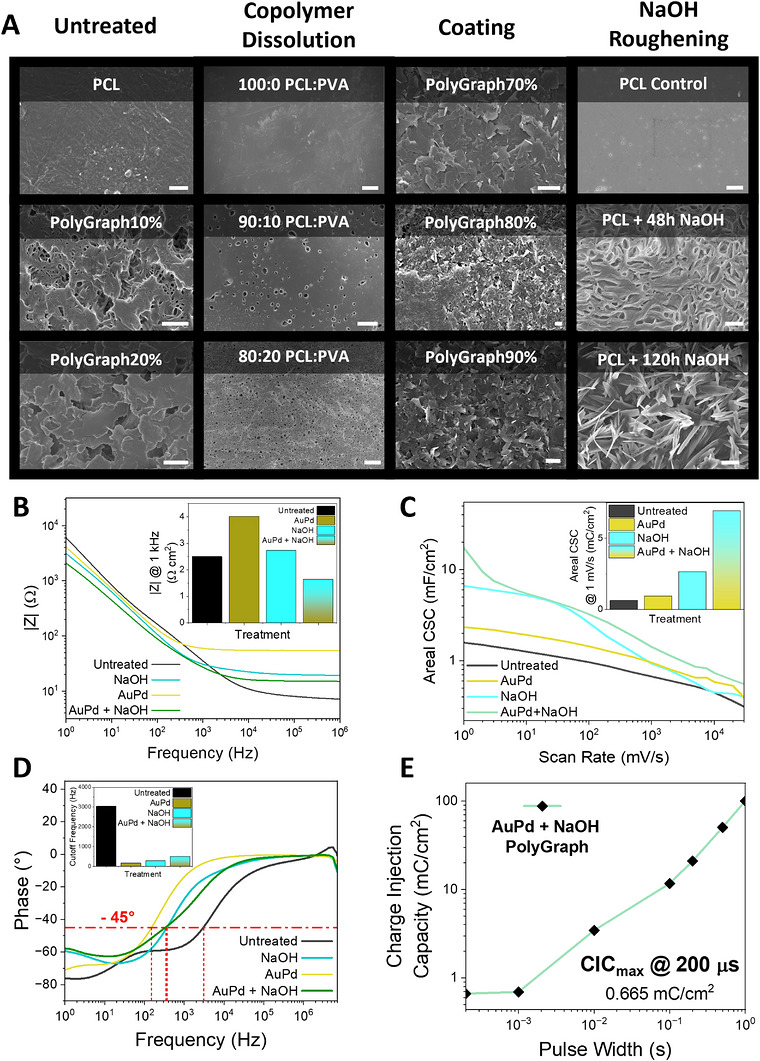
Electrical optimization & characterization of PolyGraph: (A) SEM images of PCL and PolyGraph following application of roughening treatments, from left to right: untreated PolyGraph, copolymer dissolution with PVA, spray coating with high‐loading PolyGraph, NaOH roughening. Scale bars 2 µm, except for copolymer dissolution, which are 100 µm. (B) Electrochemical impedance spectroscopy, with Bode plots showing reduced impedance after AuPd coating and NaOH treatment of PolyGraph10%. Inset: areal impedance at 1 kHz. (C) Cyclic voltammetry showing significantly increased charge storage capacity following NaOH and AuPd treatment. Inset: CSC at 1 mV/s scan rate. (D) Cut‐off frequency analysis derived from Bode phase plots, with significant decrease observed following surface treatment. (E) Voltage transients analysis determining charge injection capacity (CIC), with surface‐treated PolyGraph10% achieving a CIC of 0.665 mC/cm^2^ for a 200 µs pulse.

Some of the most critical factors in electrode design for biomedical applications are cytocompatibility and immune response, as they dictate the safety and lifetime of implanted devices [112]. We therefore investigated the ability of neuronal cells to grow on PolyGraph without inducing adverse responses. First, the ability of human neuroblastoma‐derived SH‐SY5Y cells to grow and colonise PolyGraph substrates was assessed. Neurons grown on all tested films demonstrated increases in metabolic activity and DNA content after 3 days of growth, indicative of cell proliferation and surface colonization, while showing no significant changes in metabolic activity (Figure 4A) or DNA content (Figure 4B) compared to control substrates. Next, to evaluate biocompatibility with a more physiologically relevant cell type, human induced pluripotent stem cell (iPSC)‐derived neurons were seeded on the PolyGraph films and allowed to grow for 14 days. PolyGraph10% supported the extension of neurites as well as significantly increased cellular coverage of the films (Figure 4C,D; Figure S7), potentially due to enhanced surface roughness and substrate conductivity [107]. PolyGraph10% also showed trends toward increased metabolic activity and maximum neurite length (Figure S8A,B). Interestingly, PolyGraph20% reduced neuronal populations (Figure 4D), suggesting a critical graphene loading exists beyond which adverse effects may arise due to increased exposure to nanosheet edges (Figure 5A) and resulting mechanical stress on cells [105].

Mouse primary cortical neurons cultured on PolyGraph confirmed its broad biocompatibility, even at higher loadings (Figure S9). These findings emphasize the importance of optimizing the graphene loading, as it is difficult to predict the biological behavior of nanomaterials in different configurations due to complex physical and cellular interactions.

#### 2.4.2 Glial Response

The foreign body response, driven by reactive responses of CNS‐resident astroglial cells, is another critical determinant of long‐term electrode performance, as even low levels of fibrotic scarring can severely limit device lifetime and function [113]. We assessed the response of naïve iPSC‐derived astrocytes to PolyGraph, finding that PolyGraph10% supported healthy stellate morphologies with long processes (Figure 4C) [114]. ELISA revealed no significant changes in the release of anti‐inflammatory IL‐10 or pro‐inflammatory IL‐6 from cells grown on PolyGraph compared to collagen, indicating an absence of cell polarization (Figure 4E,F).

A slight trend toward reduced LDH release on PolyGraph films was observed at day 7, with no impact on the metabolic activity of the astrocytes (Figure S8C,D). Together, these data indicate that PolyGraph10% is capable of supporting astroglial growth without inducing damaging foreign body responses from physiologically relevant cells. Combined with its mechanical, electrical, and biological advantages, these findings position PolyGraph10% as a flexible, biocompatible platform for long‐term neural interfaces, and justify its selection for further device development.

### 2.5 Electrochemical Characterization of PolyGraph

With the biocompatibility of PolyGraph10% with key neuronal and glial cell types established, focus was shifted to its electrochemical performance, to assess its suitability for neural stimulation and recording. Key parameters – surface impedance (*Z*), charge storage capacity (CSC), cut‐off frequency (*f_cut‐off_*) and charge injection capacity (CIC) – were assessed in untreated films, and following surface modification. These modifications were employed to enhance electrochemical performance by increasing the electrochemical surface area (ESA) [109, 110] and decreasing the impedance (Figure 5A). These parameters impact the signal‐to‐noise ratio (SNR), efficiency of stimulation, resolution of recording, and other key outputs [112], and can be tuned by material choice, post‐treatment, and device morphology [115].

Untreated PolyGraph10% exhibited a surface impedance of 2.5 Ω cm^2^ at 1 kHz (Figure 5B) and a charge storage capacity (CSC) of 1.6 mC/cm^2^ at a scan rate of 1 mV/s (Figure 5C), with a cutoff frequency of 3,040 Hz. The electrochemical potential window, assessed by chronoamperometry, was ±0.5 V (Figure S10B), narrower than the thermodynamic water window (∼±0.6 V) [116]. These values, while within literature ranges for impedance and CSC (0.01–1 Ω cm^2^ & 0.2–100 mC/cm^2^ [117, 118, 119]), and sufficient for large electrodes and stimulatory applications, fall short of the requirements for high‐resolution neural recording [18, 120].

Several post‐treatments were tested (Figure 5A). Channel formation in PCL using polyvinyl alcohol (PVA) as a sacrificial copolymer increased porosity, but proved inconsistent. Spray coating with high‐loading PolyGraph did not significantly alter CSC (Figure s12 A), and the reduction in polymer binding led to a risk of electrode delamination. Both approaches were therefore excluded from further development.

Sodium hydroxide (NaOH) treatment, a well‐established technique for roughening PCL [121, 122, 123], dramatically improved electrode performance. CSC increased 11‐fold to 17.3 mC/cm^2^ (Figure 5C), corresponding to a volumetric capacitance of 787.4 mF/cm^3^ (Figure S12B). This increase is likely due to increased ESA following surface roughening, which has been shown to significantly influence the CSC [109, 110, 124, 125].

SEM imaging confirmed the roughened surface, and literature suggests such surface modification may also enhance cellular adhesion and survival [106, 108, 121, 123]. To further reduce impedance, a thin AuPd coating was applied [126]. This treatment decreased the impedance of the composite materials significantly when used in conjunction with NaOH treatment, from 2.5 Ω cm^2^ to 1.6 Ω cm^2^ (Figure 5B), evidence of the beneficial synergistic effect of these treatments. The safe electrochemical potential window also widened to ±0.7 V (Figure S10C), supporting higher current delivery without electrolysis. Cut‐off frequency was reduced to 495 Hz (Figure 5D), ideal for action potential recording [115], and CIC measured using a three‐electrode setup reached ∼100 mC/cm^2^ for 1 s pulses, with ∼0.65 mC/cm^2^ at 200 µs (Figure 5E; Figure S13B). These values compare favorably to literature reports of CIC in neural electrodes (0.1 – 80 mC/cm^2^) [118, 127, 128, 129, 130, 131]. Limitations in accounting for pulse width and access voltage suggest these are conservative estimates [115].

These findings demonstrate that NaOH and AuPd treatments synergistically enhance the electrochemical performance of PolyGraph10% electrodes, enabling safe and efficient neural stimulation and high‐resolution recording. This opens new pathways for developing minimally invasive neural devices using electrochemically optimized PolyGraph, capable of addressing the complex demands of clinical and research applications. Building on this, materials with proven biocompatibility and electrochemical performance hold significant potential across bioelectronics, including in small site‐size neural interfaces, as explored in the next sections.

### 2.6 Microneedle‐Based Neural Interface Fabrication

**FIGURE 6:**
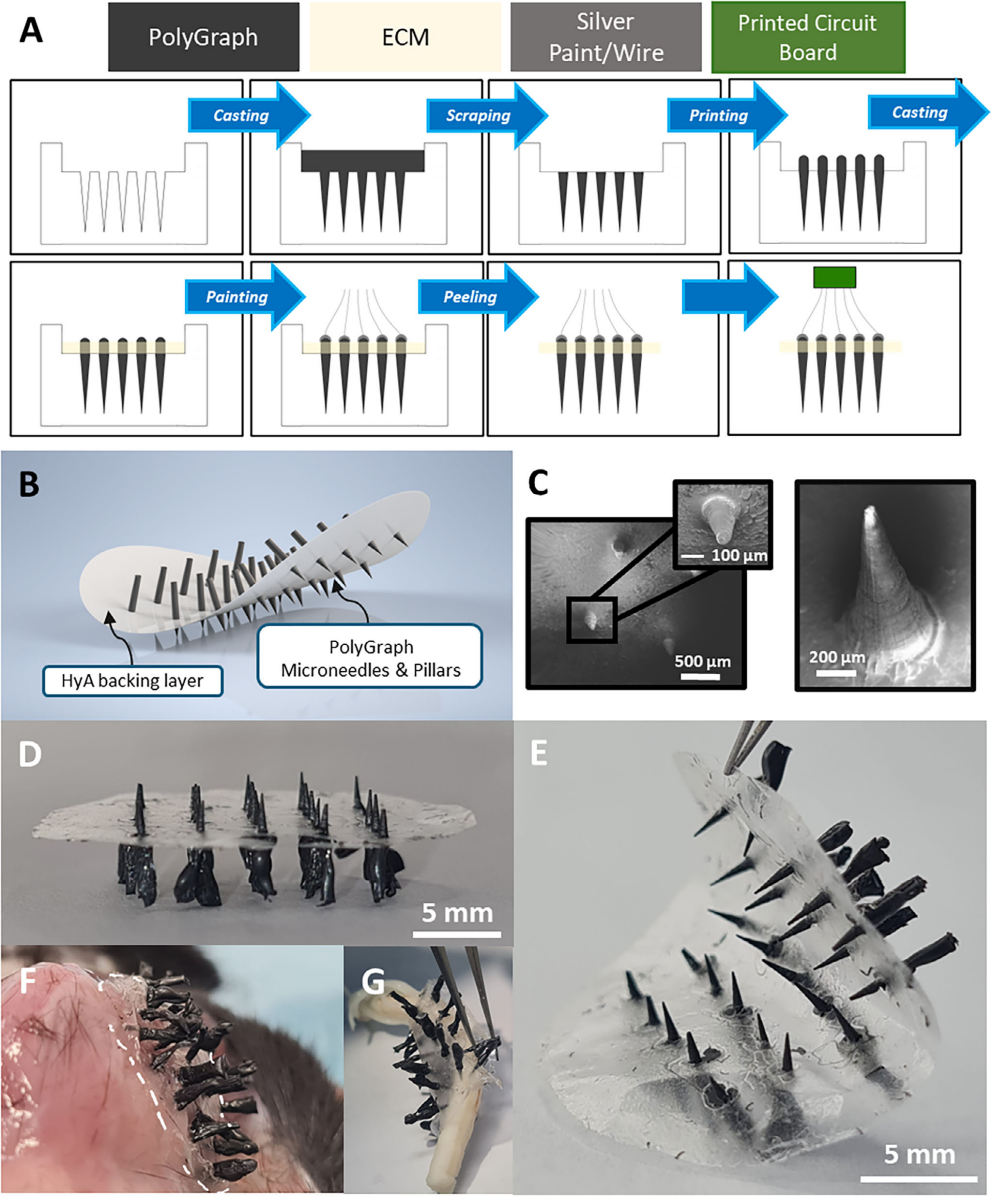
Fabrication of flexible, electrically isolated microneedle arrays: (A) Schematic of PolyGraph microneedle array fabrication workflow, leverage composite processability for dry casting and 3D printing. (B) Render of final device, showing PolyGraph microneedles and pillars anchored within a flexible hyaluronic acid (HyA) backing layer. (C) SEM images of individual PolyGraph microneedles at various magnifications, highlighting sharp tips and uniform geometry. (D–G) Optical images of the fabricated flexible, isolated microelectrode array: D), isolated array, E) demonstrating mechanical flexibility of backing, F) penetrating mouse dorsal tissue (white outline indicates hyaluronic acid backing), and G) wrapping around a rat spinal cord. Scale bars as shown.

Having successfully developed and optimized PolyGraph10% composites with enhanced electrochemical properties via NaOH roughening and AuPd coating, as a proof of concept, we next utilized these PolyGraph10% composites to fabricate a flexible microneedle‐based neural interface. Effective high‐resolution neural interfacing requires miniaturization, flexibility and high channel count [18], typically achieved using expensive fabrication techniques such as photolithography and chemical vapor deposition [132]. Here, we demonstrate a scalable, low‐cost mold‐based fabrication protocol (Figure 6A), yielding flexible microneedle arrays with individually isolated electrodes. These electrodes are connected by a hyaluronic acid extracellular matrix (ECM) backing that mimics the primary matrix component of the surrounding CNS [31, 68], improving integration with the brain surface and decoupling implant micromotion from that of the brain (Figure 6B).

PolyGraph10% microneedle arrays were melt‐cast using custom silicone molds, then combined with 3D‐printed PolyGraph10% to produce isolated needle‐pillar electrode pairs (Figure S14A,B). SEM (Figure 6C) and white light interferometry (WLI, Figure S14C) confirmed high‐quality microneedles with ∼50 µm tips. The 3D printability of PolyGraph10% also enabled custom scaffold and bioelectronic circuit fabrication (Figure S14D–F).

To minimize mechanical mismatch and prevent fibrotic encapsulation, the microneedles were linked via a thin, flexible hyaluronic acid backing. This freestanding, highly flexible microelectrode array (Figure 6D,E) is capable of conforming to complex tissue surfaces including mouse muscle (Figure 6F; Figure S15B) and rat spinal cord (Figure 6G; Figure S15A,C), unlike traditional rigid electrodes [112, 133]. We anticipate that these device components, upon their expected degradation in physiological conditions, will be safely excreted via typical glymphatic pathways without inducing cytotoxicity [134, 135].

Mechanical testing confirmed the backing's softness, with forces remaining below the 0.01 N detection limit of our tensile tester, approaching a modulus comparable to native CNS tissue (<1 kPa) [31]. This softness is critical for reducing micromotion‐induced chronic inflammatory responses upon implantation.

These advances establish PolyGraph10%‐based microneedle arrays as a scalable, flexible, and biocompatible platform for minimally invasive, high‐resolution neural interfaces, capable of addressing both central and peripheral targets.

### 2.7 Bidirectional Recording and Stimulation in Ex Vivo Brain Tissue Using PolyGraph10% Electrodes

**FIGURE 7:**
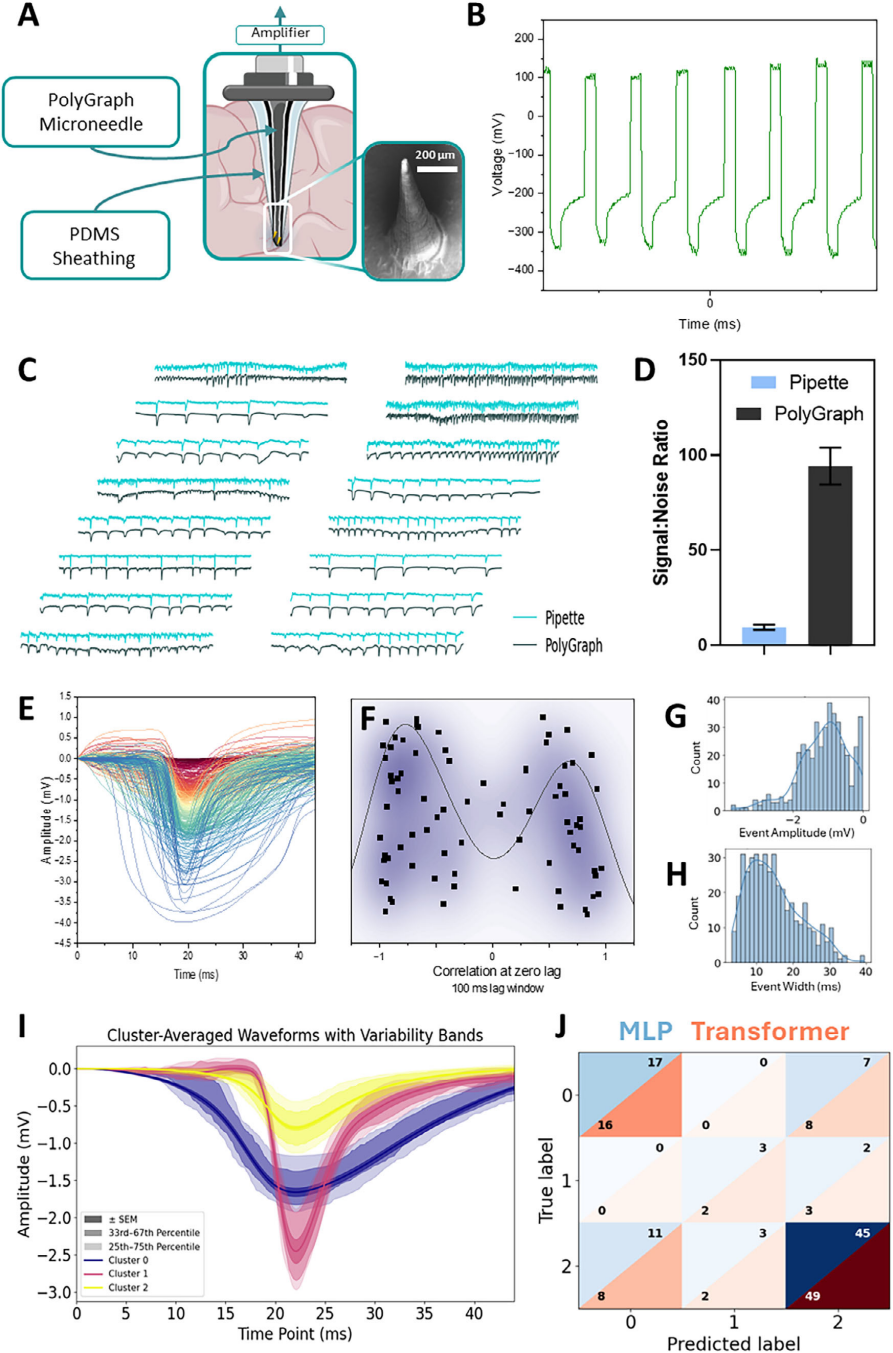
Electrophysiological recording using PolyGraph microelectrodes: (A) Schematic of a PolyGraph microneedle electrode sheathed in PDMS to reduce active electrode site size. Inset: SEM image of sharp microneedle tip. (B) Biphasic stimulation waveform delivered using PolyGraph microneedle via IonOptix pulse generator and recorded via oscilloscope. (C) Neuronal activity traces from simultaneous recordings with a standard pipette electrode (blue) and a PolyGraph microelectrode (black), showing correlated neuronal activity. (D) Signal‐to‐noise ratio (SNR) of recorded traces, with PolyGraph electrodes yielding SNR suitable for high‐resolution recording. (E) Overlay of aligned neuronal events recorded by PolyGraph, displaying typical local field activity event morphology. (F) Correlation analysis between events recorded by pipette and PolyGraph electrodes, showing strong agreement. (G, H) Event feature distributions for PolyGraph recordings: amplitude (G) and width (H). (I) Cluster‐averaged waveforms with variability bands from k‐means clustering of PolyGraph event traces, revealing distinct neuronal event types. (J) Confusion matrix for MLP neural network and simple transformer‐based classifiers, showing high classification accuracy of events into k‐means derived clusters.

Finally, to demonstrate the potential for this PolyGraph10% device as a functional bidirectional neural interface, we assessed its ability to deliver stimulation regimes in physiological buffer, and to record neuronal activity in ex vivo acute murine brain slices. Individual microneedles, fabricated as described above and insulated with PDMS to expose only the tip [18, 120] (Figure 7A; Figure S16), were integrated into an electrophysiological recording system. Compressive testing confirmed that the microneedles could withstand typical microneedle insertion forces into brain tissue (0.7 – 40 mN) [136], with a maximum compressive force of 0.36 N sustained before buckling (Figure S14G).

First, PolyGraph microneedles successfully delivered biphasic stimulation pulses in phosphate‐buffered saline, demonstrating charge injection capability in a physiologically relevant environment (Figure 7B; Figure S17B). Next, we conducted electrophysiological recordings using PolyGraph microneedle electrodes, with classical borosilicate microneedles as control electrodes (Figure S17A). PolyGraph microneedles captured electrical activity, encouraged by potassium‐rich artificial cerebrospinal fluid (high‐K aCSF) perfusion, in murine brain slices, with waveforms characteristic of extracellular neuronal events (Figure 7C,E; Figure S18).

Signal‐to‐noise ratio (SNR) analysis revealed enhanced performance compared to standard pipette electrodes (Figure 7D), and cross‐correlation confirmed consistent detection of matching neuronal activity with minimal temporal lag, due to spatial separation (Figure 7F; Figure s19). Recorded events exhibited diverse amplitudes and durations (Figure 7G,H; Figures S20 and s21), reflecting physiological events such as population spikes from groups of pyramidal neurons, field excitatory postsynaptic potentials (fEPSPs) and other network dynamics [137, 138, 139].

To extract additional physiological insights, *k*‐means clustering was applied to recorded event features to classify them according to their shape. This revealed three distinct waveform groups, visualized by their representative average traces with variability bands (Figure 7I; Figures S22 and S23). These clusters likely represent events from a mixture of different origins (e.g. population spiking, fEPSPs, fIPSPs, etc.), that enclose various phenomena (population synchronicity, excitation/inhibition balance, etc.), offering a valuable layer of physiological context for brain‐computer interface (BCI) applications.

With a view to achieving on‐chip event classification, a simple multi‐layer perceptron and linear transformer classifier were trained on the clustered events. Both models achieved high classification accuracy (Figure 7J), enabling automated, real‐time discrimination of neuronal events, allowing for interpretation and closed‐loop modulation of neuronal signaling in future devices for both BCI applications and the treatment of diseases such as epilepsy and Parkinson's disease [9, 10].

These results demonstrate PolyGraph10%’s capacity for high‐resolution, bidirectional interfacing, showcasing current delivery, neuronal activity recording, and machine learning‐based classification. This establishes PolyGraph10% as a next‐generation neural interfacing platform, for closed‐loop neuromodulation and real‐time BCI applications.

Building on PolyGraph as a neural interfacing platform material, several avenues for future development remain. Chronic in vivo studies will be critical to evaluate long‐term biocompatibility and integration with brain tissue, mechanical stability, and high‐resolution recording performance in physiological environments [140, 141, 142]. Leveraging alternative polymer matrices such as shape‐memory, stimuli‐responsive, elastomeric, and hydrogel materials will expand the range of accessible applications, and unlock complex device designs such as neural meshes and threads [18, 143, 144]. Incorporating biohybrid strategies such as cell‐laden hydrogel coatings could further mitigate immune responses and enhance tissue integration [64]. Alternative surface treatments, including conductive polymer coatings (e.g. PEDOT:PSS) [145, 146] and nanoscale texturing [147, 148, 149], may extend electrochemical performance for both stimulation and recording applications. At the device level, scaling to high‐density, multiplexed arrays with individually addressable channels across a wide area of coverage would enable next‐generation closed‐loop systems [144]. Finally, the integration of machine learning‐based classification demonstrated here offers a promising route toward real‐time, on‐chip analytics, showing that these advanced materials are compatible with state‐of‐the‐art signal processing and analysis techniques for BCIs. Together, these advances stand to position PolyGraph as a transformative platform for minimally invasive, chronic neural interfacing across both the central and peripheral nervous systems, with far‐reaching implications for clinical and neurotechnological applications.

## 3 Conclusions

The pursuit of soft, flexible, high‐performance neural interfaces demands electroconductive materials that balance biocompatibility, stability, and low neuroinflammatory potential with electrical performance, processability, and flexibility. This work introduces PolyGraph, a novel PVP‐stabilized graphene‐polycaprolactone nanocomposite optimized for neural interfacing, combining flexibility, excellent electrochemical performance, and physiologically relevant biocompatibility. Surface engineering via NaOH roughening and AuPd coating yielded low impedance (∼1.6 Ω cm^2^ @ 1 kHz) and high charge injection capacity (11.7 mC/cm^2^ for a 100 ms pulse), key benchmarks for neural stimulation and recording. These composites were cast and 3D printed into freestanding microneedle arrays, connected using a bioresorbable hyaluronic acid‐based backing to form a soft, multichannel electrode for minimally invasive, high‐resolution neural interfacing. Bidirectional functionality was validated ex vivo, with PolyGraph10% microneedles delivering controlled stimulation and recording of extracellular neuronal activity from murine brain slices, alongside machine learning‐based classification of neuronal events. Taken as a whole, these advances position PolyGraph10% as a versatile platform for soft bioelectronics, poised to drive progress in neuroprosthetics, regenerative medicine, and next‐generation brain‐computer interfaces.

## 4 Methods

### 4.1 Exfoliation of Graphene

#### 4.1.1 Gelatin Graphene Exfoliation – GelGr

Bovine gelatin (30 mg/mL, Sigma‐Aldrich, Ireland) was dissolved in deionized (DI) water, and mixed with graphite (50 mg/mL, Graphexel Ltd, UK). The dispersion was exfoliated by probe ultrasonication (VCX‐750, Sonics, USA, amplitude 80%, max power 200 W, cycle 6 s on/2 s off) for 72 h at room temperature (RT). Liquid cascade centrifugation (LCC) [150] at 500 rpm and 1000 rpm removed unexfoliated graphite and obtain size selected graphene. The dispersion was then repeatedly washed at 2000 rpm, redispersing in DI water each time until gelatinous bubbles no longer formed upon shaking. The final concentration was then determined by vacuum filtration.

#### 4.1.2 Trypsin Digestion of Gelatin Graphene – TrypGr

To enhance conductivity by thinning the gelatin coating on graphene, 20 mL of gelatin‐graphene and 10 mL of trypsin solution (Sigma‐Aldrich, Ireland) were stirred on a hot plate at 50 °C for 8 h. The resulting dispersion was centrifuged at 6000 rpm for 90 min, and the sediment was washed twice in DI water at 6000 rpm to remove excess trypsin and peptides.

#### 4.1.3 PVP (polyvinylpyrrolidone) Graphene Exfoliation – PVPGr

Graphite (40 mg/mL, Graphexel Ltd, UK) was dispersed in 80 mL isopropanol (99.9% HPLC IPA, Sigma‐Aldrich, Ireland) with 2 mg/mL of PVP powder (Sigma‐Aldrich, Ireland). The dispersion was then sonicated (VCX‐750, Sonics, USA) for 12 h at 55% amplitude, on a 6 s on/2 s off cycle. Size selection was carried out by LCC between 1000 and 6000 rpm. To remove excess PVP or exchange solvents, the dispersion was centrifuged four times at 6000 rpm for 90 min. The below exfoliation parameters were varied to determine the optimal parameters for conductivity and yield of the PVPGr exfoliation:

**TABLE 1:** Exfoliation parameters for PVP graphene optimization testing: Description of exfoliation parameters tested during optimization of polyvinylpyrrolidone (PVP) based exfoliation protocol.

Method	PVP Concentration (mgmL)	Graphite Concentration (mg/mL)	Solvent	Sonication Time (h)	Sonication Power (W)
**PVPGr A**	2	50	IPA	72	250
**PVPGr B**	2	50	IPA	8	375
**PVPGr C**	2	50	IPA	12	375

#### 4.1.4 Surfactant Exfoliation of Graphene – SCGr

Graphite (40 mg/mL) was dispersed in 80 mL DI water with sodium cholate surfactant (6 mg/mL, SC, Sigma‐Aldrich, Ireland). To pretreat the dispersion and remove impurities, the dispersion was probe ultrasonicated for 1 h, (55% amplitude, max power 750 W, cycle of 6 s on/2 s off). The dispersion was then centrifuged at 6000 rpm for 60 min, and the supernatant was discarded. The sediment was redispersed in 80 mL DI water, and 2 mg/mL SC was added. The dispersion was then sonicated for 8 h at 55% amplitude, on a 6 s on/2 s off cycle. Size selection was carried out by LCC between 1000 and 6000 rpm. To remove excess SC or exchange solvents, the dispersion was centrifuged four times at 6000 rpm for 90 min.

### 4.2 Physical Characterization of Graphene

#### 4.2.1 Scanning Electron Microscopy (SEM) to Assess Material and Device Morphology

Samples were sputter coated with a 5 nm layer of an 80:20 gold‐palladium mixture using a Cressington 108 auto sputter coater. Imaging at varying magnifications was performed on a Zeiss Ultra FE‐SEM using the InLens detector at 3 kV accelerating voltage, 30 µm aperture size, and 5 mm working distance.

#### 4.2.2 Thermogravimetric Analysis (TGA) to Assess Degree of Polymer Coating on Graphene

TGA was performed using a Q50 thermogravimetric analyzer (TA Instruments, USA). Samples were heated in air at 10 °C min^−1^, from 25 °C to 1000 °C.

#### 4.2.3 Ultra‐Violet/Visible Light (UV–vis) Spectroscopy to Investigate Optical Properties of Graphene Dispersion

UV–vis spectroscopy was conducted between 220 and 1600 nm using a Perkin‐Elmer Lambda 1050+ photospectrometer with an integrating sphere, to collect scattering, absorption, and extinction spectra.

#### 4.2.4 Raman Spectroscopy to Investigate Graphene Defects and Lateral Size

IPA‐graphene dispersions were dried onto silicon substrates at 100 °C. Raman spectra were acquired using a WITec RISE Raman system with a 633 nm laser, under ambient conditions with a spectral resolution of 2.5 cm^−1^ using a 600 g/mm grating.

#### 4.2.5 Atomic Force Microscopy (AFM) to Assess Graphene Dimensions

AFM was performed on a Bruker Multimode 8 microscope. Diluted graphene inks (1:100 in IPA) were drop‐cast onto Si/SiO_2_ substrates and imaged using OLTESPA R3 cantilevers in ScanAsyst mode. Statistical data were derived from the analysis of 173 flakes using Gwyddion SPM software [151]. The lateral dimensions were calculated by computing the square root of the product of the flake length and width.

#### 4.2.6 Contact Angle Measurements to Assess Hydrophilicity of Nanomaterial Composites

DI water contact angles were measured on 10 mm PVPGr/PCL discs using an OCA 25 contact angle goniometer (DataPhysics Instruments, USA), dispensing 5 µL DI water per drop.

#### 4.2.7 Mechanical Testing to Measure Stress‐strain Curves and Buckling Strength

Mechanical properties were derived from stress‐strain curves obtained using a Zwick Z0.5 Proline tensile tester fitted with 5 N and 100 N load cells, at a strain rate of 0.01 %/s. Samples strips were clamped and tested in uniaxial tension.

#### 4.2.8 Optical Profilometry to Assess Surface Roughness of Nanomaterial Composites

3D surface profiles were obtained using a Profilm3D Optical Profiler (Filmetrics) in white‐light interferometry (WLI) mode with a 50× Nikon DI objective lens. Calibration was performed using a gold thin film on Si/SiO_2_ with a 50 nm step height confirmed by AFM. Profiles were levelled using a three‐point levelling method, and step heights were calculated with the histogram step‐height tool. The profile was smoothed using the “remove outliers” function in ProfilmOnline.

### 4.3 PCL‐Graphene Composite (PolyGraph) Manufacture

Graphene pellets of known mass were sedimented from IPA by centrifugation at 6000 rpm for 90 min. The supernatant was discarded and replaced with polycaprolactone (PCL, M_w_ = 25 kDa) dissolved in an organic solvent (dichloromethane or chloroform, 100 mg/mL). The composite slurry was then redispersed using a tapered probe ultrasonicator for 2 h at 30% amplitude. This slurry was then cast and dried into thin layers in Teflon molds. This process is shown in Figure 3A.

### 4.4 Cell Culture for Biocompatibility and Stimulation Testing

#### 4.4.1 NSC‐34 Mouse Motor Neurons

NSC‐34 cells (Cedarlane Laboratories) were cultured in Dulbecco's Modified Eagle Medium (DMEM, Sigma‐Aldrich) supplemented with 10% fetal bovine serum (FBS, Biosera, Ireland), 1% Penicillin/Streptomycin (P/s, ScienCell, USA) and 4 mM L‐glutamine (Sigma‐Aldrich, USA).

#### 4.4.2 SH‐SY5Y Human Neuroblastoma Cells

SH‐SY5Y cells (CRL‐2266, ATCC) were cultured in a 1:1 mixture of Eagle's Minimum Essential Medium and F12 Medium (Sigma‐Aldrich, Ireland), supplemented with 10% FBS, 1% P/s, and 4 mM L‐glutamine. For neuronal differentiation, cultures were switched to Neurobasal medium (Gibco, UK) with 2% B27 (Gibco, UK), 1% GlutaMAX (Sigma‐Aldrich, Ireland) and 26.5 µM all‐trans retinoic acid (atRA, Sigma‐Aldrich, Ireland).

#### 4.4.3 iPSC‐Derived Neuronal Cells

iPSC neural precursor cells differentiated into neurons over 35 days as described previously [152]. Cells were cultured in neural maintenance media (NMM): DMEM/F12 & Neurobasal medium (1:1), 1% P/s, 1% GlutaMAX, 1% MEM non‐essential amino acids (NEAA), 0.2% N21 (Biotechne, UK), 1% B27 and 75 µM 2‐mercaptoethanol (ThermoFisher, UK). For passaging, cells were detached using accutase (4–6 min, RT), centrifuged (1000 rpm, 3 min), and replated on Celltrex‐coated (1:100, R&D Systems, UK) 6‐well plates.

#### 4.4.4 iPSC‐derived Astrocytes

iPSC astrocyte progenitor cells [152] were differentiated into astrocytes over 80 days using established protocols [153, 154]. Cells were cultured in Serio EL medium [155], consisting of Advanced DMEM‐F12 (Gibco, UK), with 1% P/s, 1% GlutaMAX, 1% NEAA, 1% N21, 0.2% B27, 20 ng/mL human epidermal growth factor (EGF) and 20 ng/mL human leukaemia inhibitory factor (LIF) (Peprotech, UK).

#### 4.4.5 Primary Neurons

E18 mouse cortices were removed post‐mortem (Ethics Approval REC202005013) and placed in cold 0.1 M PBS. Cells were dissociated in a solution containing papain (40 U/mL, Worthington, USA), deoxyribonuclease I (100 U/mL, Worthington, USA), and 30 mM glucose in Hank's Balanced Salt Solution (Sigma‐Aldrich, Ireland), for 45 min at 37°C, 5% CO_2_. 1 mL of plating media (Neurobasal Plus, 2% B27 Plus, 0.5 mM GlutaMAX, and 10% Heat Inactivated Horse Serum (Sigma‐Aldrich, Ireland)) was then added. The tissue was dissociated by gentle trituration with fire‐polished Pasteur pipettes (Fisher Scientific). Neurons were counted using a haemocytometer and seeded at 5×10^5^ cells per droplet on each film, followed by overnight incubation at 37 °C and 5% CO_2_. The next day, wells were flooded with 3 mL of complete media (plating media without horse serum), and media was changed every two days (1 mL per well).

### 4.5 Biocompatibility Assessment in 2D Culture

#### 4.5.1 Cells Grown with Nanomaterial in Suspension with Culture media

Graphene dispersions in IPA were centrifuged at 6000 rpm for 90 min, and the supernatant was replaced by fresh deionized water. This step was then repeated twice to remove any residual IPA, followed by sterilization via autoclaving. Treatment media (40 and 80 µg/mL) was prepared in the relevant cell culture media. Cells were seeded at 2×10^4^ cells/well in growth media and cultured for 24 h before replacing it with treatment media containing graphene. Media replacement was carried out every 3 days.

#### 4.5.2 Cells Grown on Films of Nanomaterial Composite

10 mm PCL‐graphene discs mounted in Cell Crowns (Scaffdex, Sigma‐Aldrich, Ireland) were sterilized using ethanol, UV exposure (30 min), and dip‐washing in 10% P/s. Cells were seeded at 1×10^4^ cells/film in a small drop of growth media and allowed to attach for 1 h at 37 °C, before flooding the well with media. Media replacement was carried out every 3 days. Metabolic activity and DNA content were measured at designated timepoints following manufacturer protocols.

#### 4.5.3 Immunostaining to Assess Cellular Morphology

At specific time points, samples were washed in PBS and fixed with either 10% formalin (Sigma‐Aldrich, Ireland) or 4% paraformaldehyde (PFA, Fisher‐Scientific, Ireland) for approximately 15 min at RT, followed by three PBS washes. To assess mature neuronal morphology, samples were incubated overnight at 4 °C with rabbit anti‐β Tubulin III primary antibody (1:1000, Sigma‐Aldrich) in PBS, with 0.1% Triton‐X100 and 1% bovine serum albumin. The following day, after three PBS washes, samples were incubated with Alexa Fluor 555 Goat Anti‐Rabbit IgG (1:1000, Invitrogen, UK) for 2 h at RT. To visualize cytoskeletal structure of both neuronal and glial cells, samples were incubated overnight at 4 °C with fluorescein isothiocyanate (FITC)‐labeled phalloidin (1:1000, Sigma‐Aldrich, Ireland). After three PBS washes, nuclei were counterstained with DAPI (1:1000) in PBS with 0.1% Triton‐X100, and samples mounted on slides using Fluoroshield (Sigma–Aldrich). Imaging was performed using a Zeiss AxioObserver fluorescent microscope (Carl Zeiss Ltd., USA), and images analyzed in FIJI (ImageJ 1.52p).

### 4.6 Treatment to Enhance Electrochemical Performance of PolyGraph Composites

The modulation of the electrochemical performance of PolyGraph composites was tested using a variety of techniques (Figure S11):

#### 4.6.1 Copolymer Dissolution

To increase composite porosity and specific surface area, 0–20 vol% polyvinyl alcohol (PVA) was added to PCL slurry during casting. After drying, the composite was washed in deionized water for 1 h to remove PVA, forming pores and voids.

#### 4.6.2 Spray Coating

PolyGraph composites were placed in an aerosol‐jet nanomaterial spraying setup, and an ∼100nm thick layer of PVPGr nanosheets was deposited onto the surface.

#### 4.6.3 NaOH Roughening

To increase the specific surface area by means of roughening, samples were submerged in 3 M NaOH aqueous solution for 72 h at RT, followed by rinsing with DI water.

#### 4.6.4 AuPd Coating

To decrease the surface impedance, samples were coated with a thin layer (∼10 nm) 80:20 gold‐palladium mixture, using a Cressington 108 auto sputter coater.

### 4.7 Electrical Characterization of Material and Device Performance

#### 4.7.1 Conductivity

DC conductivity was measured using a Keithley Model 2450 Sourcemeter, connected to silver paint electrodes on each end of thin strips of PCL‐graphene.

#### 4.7.2 Cyclic Voltammetry (CV), Electrochemical Impedance Spectroscopy (EIS) & Chronoamperometry (CA) to Measure Charge Storage Capacity (CSC), Impedance, and Electrochemical Potential Window

Two‐electrode coin cells were fabricated with 3 mm diameter discs of PolyGraph, using phosphate‐buffered saline (PBS) as the electrolyte, and polyethylene filters as separators (Figure S10A). CV and EIS measurements were performed on a Biologic VMP‐300 potentiostat. CV was carried out in a ±200 mV potential window at scan rates from 1 mV/s – 30000 mV/s. EIS was recorded across 1 Hz – 7 MHz at 10 mV amplitude. CA was measured in steps of 100 mV from −1 to 1 V, with the magnitude of the current transient at 1 s after stimulation extracted. Data analysis was carried out using Biologic EC‐Lab software, to extract the surface impedance (*Z*), cut‐off frequency (*f_cut‐off_*), and charge storage capacity (CSC).

#### 4.7.3 Voltage Transients Analysis to Measure Charge Injection Capacity (CIC)

Voltage transients were recorded using a Swagelok three‐electrode system with glassy carbon electrodes, a graphite reference electrode, and a carbon black/Teflon counter electrode, using PBS as the electrolyte (Figure s13 A). Chronopotentiometry delivered defined current densities, followed by measurement of the resulting voltage transients. The maximum polarization at each current density was used to calculate the maximum charge injection capacity (CIC_max_) of the tested material.

### 4.8 Microneedle Array Manufacture for Flexible BCI Prototype

Microneedle bases were designed in AutoCAD Inventor, with the desired pitch, angle, and needle length. Designs were 3D printed in clear resin using an SLA printer (Form 3/4B, FormLabs, USA, then washed in IPA for 30 min, before 60 min of UV curing at 60 °C. Polydimethylsiloxane (PDMS, Sylgard 184, Ellsworth Adhesives, Ireland) was mixed, degassed, and poured over the microneedle bases in Petri dishes to form negative molds, which were then cured overnight at 60 °C. Negative molds were then carefully peeled from the microneedle base. This process was adapted from O'Cearbhaill et al. [156, 157].

Microneedle arrays were fabricated in a multi‐stage process (Figure 6A). PolyGraph films were placed in a microneedle mold at 200 °C until softened, followed by mechanical compression with a spatula, to penetrate all needle holes. For free‐standing, non‐isolated devices, the microneedle array was peeled from the mold at this step. To produce flexible, isolated devices, excess PolyGraph was removed while still molten. Pillars of PolyGraph, dissolved in dichloromethane at 1.5 g/mL, were printed on the back of the microneedles using custom G‐code on an FDM printer (Allevi 3, Allevi, USA) and via manual extrusion using a syringe. A 5 mg/mL hyaluronic acid solution was then cast and dried between the pillars, to form a soft, conformable base. Final devices were gently released from the molds.

### 4.9 Assessment of Bidirectional Neural Interfacing Capabilities

#### 4.9.1 Ethical Declaration for Ex Vivo Experiments

All ex vivo mouse and rat tissues (mouse back tissue, rat spinal cord, and mouse brain slices), were obtained as post‐mortem byproducts from ongoing experiments in other RCSI groups which have been approved by the RCSI Research Ethics Committee (REC 1587) and under license by the Health Products Regulatory Authority (AE19127/P057), in line with institutional animal welfare policy and the 3Rs (replacement, reduction, refinement).

#### 4.9.2 Proof of Concept with Ex Vivo Tissue

**FIGURE 8:**
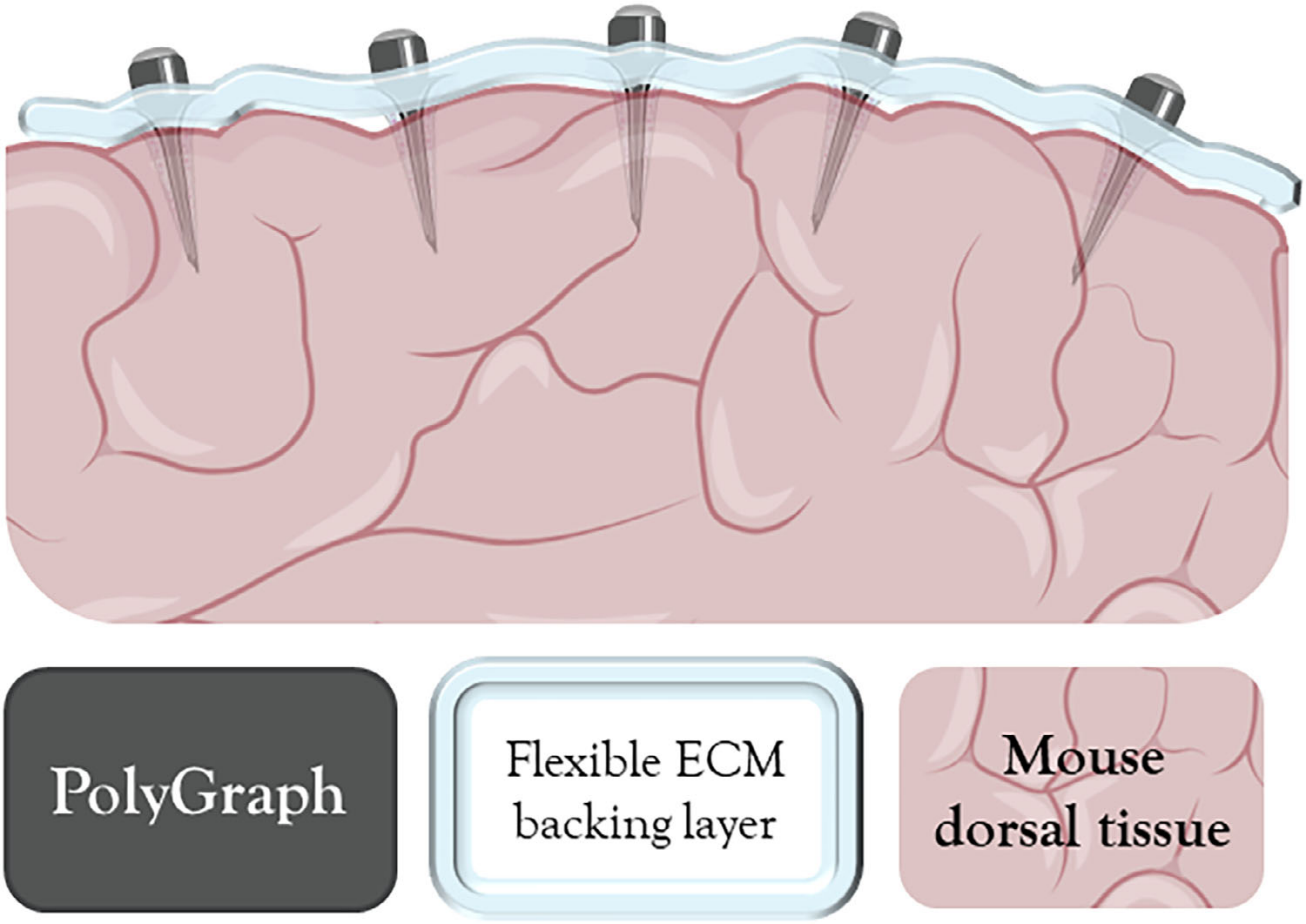
PolyGraph microneedle ex vivo tissue penetration: Schematic of penetration of ex vivo mouse dorsal tissue using a PolyGraph microneedle array supported by a flexible ECM‐based backing layer.

Final devices were carefully handled using tweezers, and positioned on the ex vivo tissue. In the case of the mouse dorsal tissue, gentle pressure was applied to the needles from the back, allowing them to penetrate the tissue. The hyaluronic acid backing, once wet, conforms and fixes the device to the tissue (Figure 8). In the case of the embalmed rat spinal cord, the device was gently wrapped around the tissue and held in place using tweezers.

#### 4.9.3 Recording of Neuronal Signals in Brain Slice Model and Delivery of Stimulation Pulses

**FIGURE 9:**
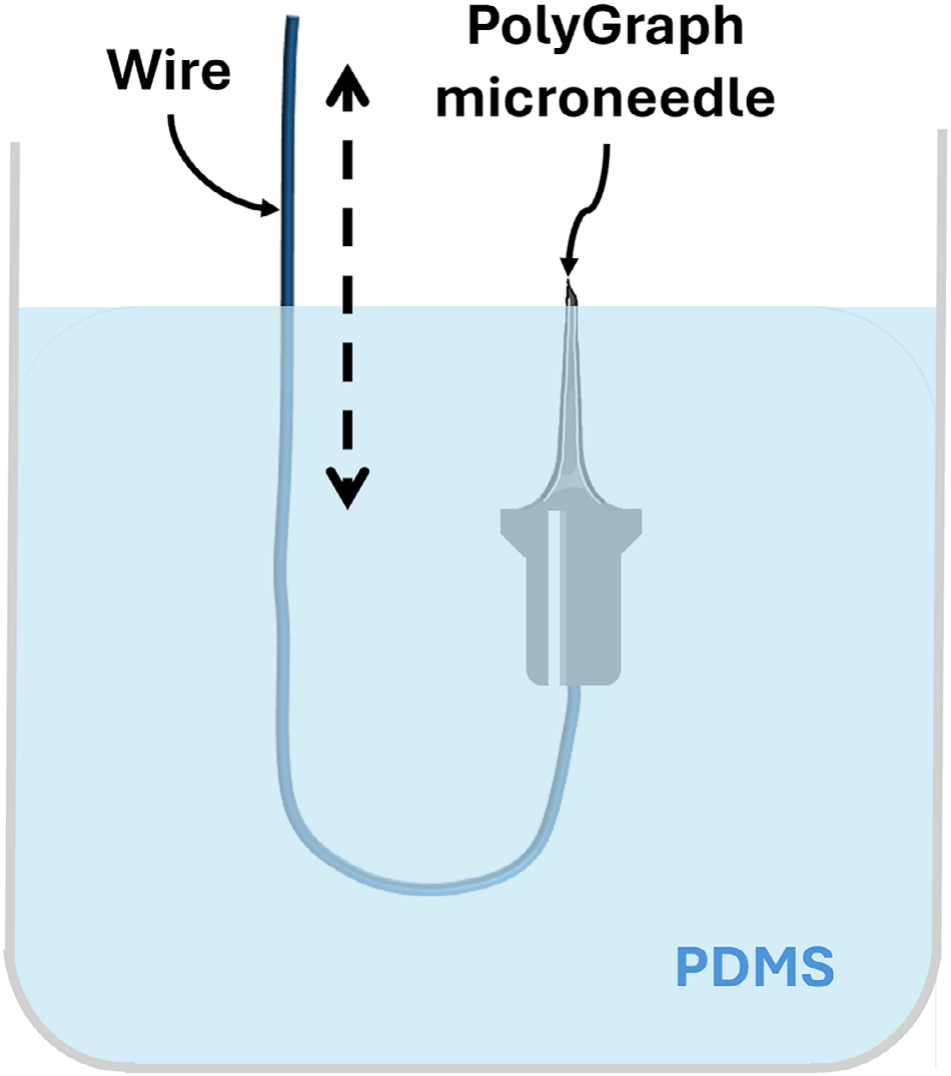
PDMS Sheathing: Schematic of PDMS sheathing of PolyGraph microneedles. Needles and their associated wires were slowly dipped into uncured PDMS, leaving only the tip exposed.

**FIGURE 10:**
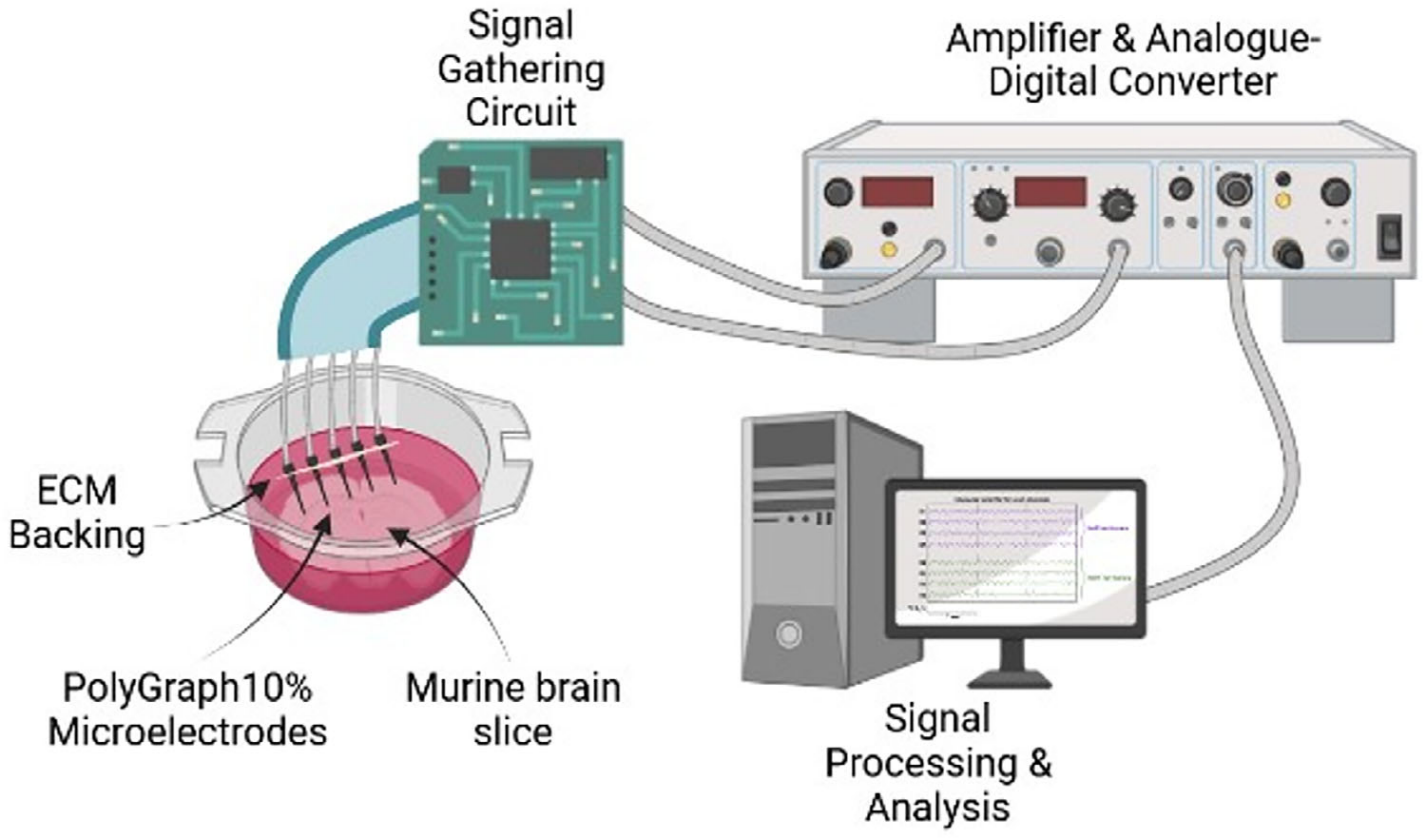
Bidirectional neural interfacing setup: Schematic of stimulation or recording from brain slice model using a flexible PolyGraph neural interface.

Individual microneedle/pillar units were detached from the molds described in Section 4.8. These units were then NaOH and AuPd treated as described in Section 4.6, and connected to wires with silver paint. To reduce electrode size and increase recording resolution, the microelectrodes were sheathed, by dipping them in liquid PDMS (Figure 9). Electrodes were wired to BNC connectors, and signals were pre‐amplified (gain = 100, SR560, Stanford Research Systems, USA), digitized (Axon DigiData 1550B, Molecular Devices) and recorded via Clampex software (Molecular Devices) at 10 kHz, with low‐pass filtering at 2 kHz. Analog signals were visualized on an oscilloscope (TBS1000C, Tektronix, USA). An AuPd‐coated wire served as the reference electrode, and a glass electrophysiological pipette electrode served as the control (Figures 10).

Acute 350 µm thick coronal brain slices were obtained using a vibratome (7000smz‐2, Campden Instruments, Loughborough, UK) from an 11‐week‐old C57/Bl6 male mouse. Briefly, the animal was deeply anesthetized (5% isoflurane) and quickly decapitated. The whole brain was subsequently removed and placed in ice‐cold oxygenated (95% O_2_, 5% CO_2_) high‐sucrose artificial cerebrospinal fluid solution (aCSF, 11 mM glucose, 1 mM MgSO_4_, 1 mM NaH_2_PO_4_, 2.5 mM KCl, 26.2 mM NaHCO_3_, 2.5 mM CaCl_2_, 119 mM NaCl and 75 mM sucrose), slices were then left to recover for at least 1 h in a chamber containing Ringer solution (126 mM NaCl, 3 mM KCl, 1.25 mM NaH_2_PO_4,_ 2 mM MgSO_4_, 2 mM CaCl_2_, 10 mM glucose and 26 mM NaHCO_3_) at RT until recording.

After recovery (>1 h), slices were transferred to a submerged recording chamber in a patch‐clamp rig constantly perfused with oxygenated Ringer solution at physiological temperature (36°C), at a flow rate of ∼7.5 mL/min. Under microscope visualization (Scientifica SliceScope), the microneedle and pipette electrodes were placed 10–100 µm apart on the stratum radiatum of the CA1 hippocampal region, and spontaneous neuronal activity was encouraged using a modified aCSF solution (0.4 mM MgSO_4_, 8 mM KCl, 300 µM picrotoxin (PTX)).

For stimulation, microneedle electrodes were connected to an IonOptix C‐Pace stimulation rig delivering biphasic current pulses, with an AuPd‐coated wire as counter electrode. The waveform of stimulation pulses was monitored on the oscilloscope via two additional AuPd‐coated wires.

#### 4.9.4 Event Analysis

Acquired recordings were notch‐filtered at 50 Hz and its first five harmonics to remove electrical interference, and low‐pass filtered at 150 Hz to remove high‐frequency noise and isolate slower LFPs. A template event, manually identified from 100 traces, and used to identify 437 distinct events in the recording from the PolyGraph electrode. Traces were processed using a custom Python analysis pipeline, which aligns and scales the waveforms prior to extracting features such as the peak value, peak width, and area under curve.

Dimensionality reduction was performed using principal component analysis (PCA) and t‐distributed Stochastic Neighbor Embedding (t‐SNE), followed by *k*‐means clustering. To test classification of unseen data, a multilayer perceptron (MLP) and linear transformer model were trained on a subset of the clustered data, and their accuracy evaluated using confusion matrices.

### 4.10 Statistical Analysis

A minimum of three experimental replicates (N) with at least three technical replicates (n) were used in all assessments. Prior determination of statistical significance, the ROUT method (Q = 10%) was used to determine the variance in the population. All data sets were subjected to Kolmogorov‐Smirnov test to assess for normality and thereafter the data tested using appropriate parametric (t‐test, one‐ and two‐way ANOVA) and non‐parametric (Mann‐Whitney U, one and two‐way Kruskall–Wallis) tests. Post‐hoc tests were performed using Tukey's multiple comparisons and Dunnett's correction where needed. All results were plotted with the standard error of mean and were considered significant when p < 0.05 and statistical significance was denoted by ^*^: ^*^ *p* < 0.05, ^**^ *p* < 0.01, ^***^ *p* < 0.001, ^****^ *p* < 0.0001. Significances are relative to control group of timepoint unless stated otherwise. All data were plotted and analyzed as means with standard error of the mean while using the software GraphPad Prism (v8.0) and OriginLab 2024.

## Funding

J. M., I. W., C. O'C., A. D., F. O'B and J. N. C. acknowledge the Research Ireland AMBER Centre for providing primary financial support to this study (SFI/12/RC/2278_P2). C. O'C. also acknowledges funding from an Anatomical Society Studentship. J. M. M., acknowledges funding from the Generation D initiative, promoted by Red.es, an organisation attached to the Ministry for Digital Transformation and the Civil Service, financed by the Recovery, Transformation and Resilience Plan through the European Union's Next Generation funds.

## Conflicts of Interest

The authors declare no conflicts of interest.

## Supporting information




**Supporting File**: adhm71176‐sup‐0001‐SuppMat.docx.

## Data Availability

The data that support the findings of this study are available from the corresponding author upon reasonable request.

## References

[1] J. A. Frank , M.‐J. Antonini , and P. Anikeeva , “Next‐generation Interfaces for Studying Neural Function,” Nature Biotechnology 37 (2019): 1013–1023. 10.1038/s41587-019-0198-8 PMC7243676 31406326

[2] Y. Wang , S. Liu , H. Wang , Y. Zhao , and X.‐D. Zhang , “Neuron Devices: Emerging Prospects in Neural Interfaces and Recognition,” Microsystems & Nanoengineering 8 (2022): 1–13. 36507057 10.1038/s41378-022-00453-4 PMC9726942

[3] M. Zhang , Z. Tang , X. Liu , and J. Van der Spiegel , “Electronic Neural Interfaces,” Nat Electron 3 (2020): 191–200.

[4] M. Wang , G. Mi , D. Shi , N. Bassous , D. Hickey , and T. J. Webster , “Nanotechnology and Nanomaterials for Improving Neural Interfaces,” Advanced Functional Materials 28 (2018): 1700905.

[5] Q. Zeng , X. Li , S. Zhang , C. Deng , and T. Wu , “Think Big, See Small—A Review of Nanomaterials for Neural Interfaces,” Nano Select 3 (2022): 903–918.

[6] J. A. Flack , K. D. Sharma , and J. Y. Xie , “Delving into the Recent Advancements of Spinal Cord Injury Treatment: a Review of Recent Progress,” Neural Regen Res 17 (2021): 283–291. 10.4103/1673-5374.317961 PMC8463999 34269189

[7] C. J. Bettinger , M. Ecker , T. D. Yoshida Kozai , G. G. Malliaras , E. Meng , and W. Voit , “Recent Advances in Neural Interfaces—Materials Chemistry to Clinical Translation,” MRS Bulletin 45 (2020): 655–668. 34690420 10.1557/mrs.2020.195 PMC8536148

[8] H. Shen , C. Fan , Z. You , Z. Xiao , Y. Zhao , and J. Dai , “Advances in Biomaterial‐Based Spinal Cord Injury Repair,” Advanced Functional Materials 32 (2022): 2110628.

[9] X. Li , Y. Song , G. Xiao , et al., “PDMS–Parylene Hybrid, Flexible Micro‐ECoG Electrode Array for Spatiotemporal Mapping of Epileptic Electrophysiological Activity from Multicortical Brain Regions,” ACS Applied Bio Materials 4 (2021): 8013–8022. 10.1021/acsabm.1c00923 35006782

[10] Y.‐X. Chen , P.‐J. Feng , G. Zhong , et al., “Piezoelectric Nanogenerators Enabled Neuromodulation Rescued Dopaminergic Neuron Loss in Parkinson's Disease,” Nano Energy 121 (2023): 109187.

[11] Y. Wang , X. Yang , X. Zhang , Y. Wang , and W. Pei , “Implantable Intracortical Microelectrodes: Reviewing the Present with a Focus on the Future,” Microsystems & Nanoengineering 9 (2023): 1–17. 36620394 10.1038/s41378-022-00451-6 PMC9814492

[12] D. Valeriani , F. Santoro , and M. Ienca , “The Present and Future of Neural Interfaces,” Frontiers in Neurorobotics 16 (2022): 953968. 36304780 10.3389/fnbot.2022.953968 PMC9592849

[13] A. F. Jackson and D. J. Bolger , “The Neurophysiological Bases of EEG and EEG Measurement: a Review for the Rest of Us,” Psychophysiology 51 (2014): 1061–1071. 25039563 10.1111/psyp.12283

[14] M. Teplan , “Fundamentals of EEG Measurement,” Measurement Science Review 2 (2002): 1–11.

[15] K. Värbu , N. Muhammad , and Y. Muhammad , “Past, Present, and Future of EEG‐Based BCI Applications,” Sensors 22 (2022): 3331. 35591021 10.3390/s22093331 PMC9101004

[16] T. Hinterberger , N. Weiskopf , R. Veit , B. Wilhelm , E. Betta , and N. Birbaumer , “An EEG‐driven Brain‐computer Interface Combined with Functional Magnetic Resonance Imaging (fMRI),” IEEE Transactions on Biomedical Engineering 51 (2004): 971–974. 15188866 10.1109/TBME.2004.827069

[17] B. Sorger and R. Goebel , “Chapter 21 ‐ Real‐time fMRI for Brain‐computer Interfacing,” in Handbook of Clinical Neurology, ed. N. F. Ramsey and J. d. R. Millán (Elsevier, 2020), 289–302. 10.1016/B978-0-444-63934-9.00021-4 32164860

[18] G. Hong and C. M. Lieber , “Novel Electrode Technologies for Neural Recordings,” Nature Reviews Neuroscience 20 (2019): 330–345. 30833706 10.1038/s41583-019-0140-6 PMC6531316

[19] Y. Ziai , S. S. Zargarian , C. Rinoldi , et al., “Conducting Polymer‐Based Nanostructured Materials for Brain–machine Interfaces,” WIREs Nanomedicine and Nanobiotechnology 15 (2023): 1895. 10.1002/wnan.1895 37141863

[20] R. Green and M. R. Abidian , “Conducting Polymers for Neural Prosthetic and Neural Interface Applications,” Advanced Materials (Deerfield Beach, Fla) 27 (2015): 7620. 26414302 10.1002/adma.201501810 PMC4681501

[21] Y. Qin , X. Qu , B. Huang , et al., “Vivo Synthesis of Metabolically Degradable π‑Conjugated Conductive Polymers Enabling Seamless Neural Interface Integration and Tissue Repair,” Advanced Functional Materials 35 (2025): 2501813.

[22] R. A. Green , N. H. Lovell , G. G. Wallace , and L. A. Poole‐Warren , “Conducting Polymers for Neural Interfaces: Challenges in Developing an Effective Long‐term Implant,” Biomaterials 29 (2008): 3393–3399. 10.1016/j.biomaterials.2008.04.047 18501423

[23] S. Gou , S. Yang , Y. Cheng , et al., “Applications of 2D Nanomaterials in Neural Interface,” International Journal of Molecular Sciences 25 (2024): 8615. 39201302 10.3390/ijms25168615 PMC11354839

[24] X. He , Y. Zhu , B. Ma , et al., “Bioactive 2D Nanomaterials for Neural Repair and Regeneration,” Advanced Drug Delivery Reviews 187 (2022): 114379. 35667464 10.1016/j.addr.2022.114379

[25] V. Shanmugam , R. A. Mensah , K. Babu , et al., “A Review of the Synthesis, Properties, and Applications of 2D Materials,” Part & Part Syst Charact 39 (2022): 2200031.

[26] S. Alam , M. Asaduzzaman Chowdhury , A. Shahid , R. Alam , and A. Rahim , “Synthesis of Emerging Two‐Dimensional (2D) Materials – Advances, Challenges and Prospects,” FlatChem 30 (2021): 100305.

[27] A. A. A. Ahmed , N. Alegret , B. Almeida , et al., “Interfacing with the Brain: How Nanotechnology Can Contribute,” ACS Nano 19 (2025): 10630–10717. 40063703 10.1021/acsnano.4c10525 PMC11948619

[28] S. S. Zargarian , C. Rinoldi , Y. Ziai , et al., “Chronic Probing of Deep Brain Neuronal Activity Using Nanofibrous Smart Conducting Hydrogel‐Based Brain–Machine Interface Probes,” Small Science 5 (2025): 2400463. 40395354 10.1002/smsc.202400463 PMC12087770

[29] C. Rinoldi , Y. Ziai , S. Zargarian , et al., “In Vivo Chronic Brain Cortex Signal Recording Based on a Soft Conductive Hydrogel Biointerface,” ACS Applied Materials & Interfaces 15, no. 5 (2022): 6283–6296, 10.1021/ACSAMI.2C17025. 36576451

[30] S. Cheng , R. Zhu , and X. Xu , “Hydrogels for next Generation Neural Interfaces,” Commun Mater 5 (2024): 99.

[31] I. Woods , C. O'Connor , L. Frugoli , et al., “Biomimetic Scaffolds for Spinal Cord Applications Exhibit Stiffness‐dependent Immunomodulatory and Neurotrophic Characteristics,” Advanced Healthcare Materials 11 (2021): 2101663. 10.1002/adhm.202101663 34784649

[32] J. W. Salatino , K. A. Ludwig , T. D. Y. Kozai , and E. K. Purcell , “Glial Responses to Implanted Electrodes in the Brain,” Nature Biomedical Engineering 1 (2017): 862–877. 10.1038/s41551-017-0154-1 PMC6261524 30505625

[33] A. Sridharan , S. D. Rajan , and J. Muthuswamy , “Long‐term Changes in the Material Properties of Brain Tissue at the Implant–tissue Interface,” Journal of Neural Engineering 10 (2013): 066001. 24099854 10.1088/1741-2560/10/6/066001 PMC3888957

[34] C.‐X. Hu , O. Read , Y. Shin , et al., “Effects of Lateral Size, Thickness, and Stabilizer Concentration on the Cytotoxicity of Defect‐Free Graphene Nanosheets: Implications for Biological Applications,” ACS Applied Nano Materials 5, no. 9 (2022): 12626–12636, 10.1021/ACSANM.2C02403. 36185165 PMC9513747

[35] M. Abdelmonem , E. L. Albert , A. Norman , E. Z. Tarmizie , S. H. Zyoud , and C. A. C. Abdullah , “Surface Functionalization of 2D MOs for Enhanced Biocompatibility and Biomedical Applications,” in Emerging Applications of Novel Nanoparticles, ed. S. Anil Bansal , V. Khanna , N. Balakrishnan , and P. Gupta , (Springer Nature Switzerland, 2024), 175–198, 10.1007/978-3-031-57843-4_7.

[36] T. Fan , L. Yan , S. He , et al., “Biodistribution, Degradability and Clearance of 2D Materials for Their Biomedical Applications,” Chemical Society Reviews 51 (2022): 7732–7751, 10.1039/D1CS01070K. 36047060

[37] M. Lotya , P. J. King , U. Khan , S. De , and J. N. Coleman , “High‐concentration, Surfactant‐stabilized Graphene Dispersions,” ACS Nano 4 (2010): 3155–3162. 20455583 10.1021/nn1005304

[38] Y. Hernandez , V. Nicolosi , M. Lotya , et al., “High‐yield Production of Graphene by Liquid‐phase Exfoliation of Graphite,” Nature Nanotechnology 3 (2008): 563–568. 10.1038/nnano.2008.215 18772919

[39] M. J. Fernández‐Merino , J. I. Paredes , S. Villar‐Rodil , et al., “Investigating the Influence of Surfactants on the Stabilization of Aqueous Reduced Graphene Oxide Dispersions and the Characteristics of Their Composite Films,” Carbon 50 (2012): 3184–3194.

[40] J. Maughan , P. J. Gouveia , J. G. Gonzalez , et al., “Collagen/Pristine Graphene as an Electroconductive Interface Material for Neuronal Medical Device Applications,” Applied Materials Today 29 (2022): 101629.

[41] A. J. Ryan , C. J. Kearney , N. Shen , et al., “Electroconductive Biohybrid Collagen/Pristine Graphene Composite Biomaterials with Enhanced Biological Activity,” Advanced Materials 30 (2018): 1706442. 10.1002/adma.201706442 29504165

[42] M. Seredych , K. Maleski , T. S. Mathis , and Y. Gogotsi , “Delamination of MXenes Using Bovine Serum Albumin,” Colloids and Surfaces A: Physicochemical and Engineering Aspects 641 (2022): 128580.

[43] C. V. Kumar and A. Pattammattel , “Chapter Eleven ‐ BioGraphene: Direct Exfoliation of Graphite in a Kitchen Blender for Enzymology Applications,” in Methods in Enzymology, ed. C. V. Kumar , (Academic Press, 2016), 225–244, https://www.sciencedirect.com/science/article/pii/S007668791600121X). 10.1016/bs.mie.2016.03.009 27112402

[44] F. Lebre , D. Hanlon , J. B. Boland , J. Coleman , and E. C. Lavelle , “Exfoliation in Endotoxin‐Free Albumin Generates Pristine Graphene with Reduced Inflammatory Properties,” Advanced Biosystems 2 (2018): 1800102. 10.1002/adbi.201800102 PMC13373802 42466014

[45] Y. Shin , X. Just‐Baringo , M. Boyes , et al., “Enhanced Liquid Phase Exfoliation of Graphene in Water Using an Insoluble Bis‐pyrene Stabiliser,” Faraday Discussions 227 (2021): 46–60. 33295354 10.1039/c9fd00114j

[46] Y. Shin , S. Vranic , X. Just‐Baringo , et al., “Stable, Concentrated, Biocompatible, and Defect‐free Graphene Dispersions with Positive Charge,” Nanoscale 12 (2020): 12383–12394. 32490468 10.1039/d0nr02689a

[47] M. Kurakula and G. S. N. K. Rao , “Pharmaceutical Assessment of Polyvinylpyrrolidone (PVP): as Excipient from Conventional to Controlled Delivery Systems with a Spotlight on COVID‐19 Inhibition,” Journal of Drug Delivery Science and Technology 60 (2020): 102046. 32905026 10.1016/j.jddst.2020.102046 PMC7462970

[48] K. M. Koczkur , S. Mourdikoudis , L. Polavarapu , and S. E. Skrabalak , “Polyvinylpyrrolidone (PVP) in Nanoparticle Synthesis,” Dalton Transactions 44 (2015): 17883–17905. 26434727 10.1039/c5dt02964c

[49] H. Yu , B. Wang , S. Zhou , et al., “Polyvinylpyrrolidone Functionalization Induces Deformable Structure of Graphene Oxide Nanosheets for Lung‐targeting Delivery,” Nano Today 38 (2021): 101151.

[50] A. B. Bourlinos , V. Georgakilas , R. Zboril , T. A. Steriotis , A. K. Stubos , and C. Trapalis , “Aqueous‐phase Exfoliation of Graphite in the Presence of Polyvinylpyrrolidone for the Production of Water‐soluble Graphenes,” Solid State Communications 149 (2009): 2172–2176.

[51] A. S. Wajid , S. Das , F. Irin , et al., “Polymer‐stabilized Graphene Dispersions at High Concentrations in Organic Solvents for Composite Production,” Carbon 50 (2012): 526–534.

[52] T. Lalire , C. Longuet , and A. Taguet , “Electrical Properties of Graphene/Multiphase Polymer Nanocomposites: a Review,” Carbon 225 (2024): 119055.

[53] S. G. Er , M. Edirisinghe , and T. A. Tabish , “Graphene‐based Nanocomposites as Antibacterial, Antiviral, and Antifungal Agents,” Advanced Healthcare Materials 12 (2022): 2201523. 10.1002/adhm.202201523 PMC11468666 36511355

[54] A. Qadir , T. K. Le , M. Malik , et al., “Representative 2D‐material‐based Nanocomposites and Their Emerging Applications: a Review,” RSC Advances 11 (2021): 23860–23880. 35479005 10.1039/d1ra03425a PMC9036868

[55] E. Malikmammadov , T. E. Tanir , A. Kiziltay , V. Hasirci , and N. Hasirci , “PCL and PCL‐based Materials in Biomedical Applications,” Journal of Biomaterials Science, Polymer Edition 29 (2018): 863–893. 29053081 10.1080/09205063.2017.1394711

[56] X. Yang , Y. Wang , Y. Zhou , J. Chen , and Q. Wan , “The Application of Polycaprolactone in Three‐Dimensional Printing Scaffolds for Bone Tissue Engineering,” Polymers (Basel) 13 (2021): 2754. 34451293 10.3390/polym13162754 PMC8400029

[57] S. Sayyar , E. Murray , B. C. Thompson , S. Gambhir , D. L. Officer , and G. G. Wallace , “Covalently Linked Biocompatible Graphene/Polycaprolactone Composites for Tissue Engineering,” Carbon 52 (2013): 296–304.

[58] M. Karbalaei Akbari , N. Siraj Lopa , M. Shahriari , A. Najafzadehkhoee , D. Galusek , and S. Zhuiykov , “Functional Two‐Dimensional Materials for Bioelectronic Neural Interfacing,” Journal of Functional Biomaterials 14 (2023): 35. 36662082 10.3390/jfb14010035 PMC9863167

[59] J. A. Fairfield , “Nanostructured Materials for Neural Electrical Interfaces,” Advanced Functional Materials 28 (2018): 1701145.

[60] D. Viana , S. T. Walston , E. Masvidal‐Codina , et al., “Nanoporous Graphene‐based Thin‐film Microelectrodes for in Vivo High‐resolution Neural Recording and Stimulation,” Nature Nanotechnology 19 (2024): 514–523, 10.1038/s41565-023-01570-5. PMC11026161 38212522

[61] M. Ferguson , D. Sharma , D. Ross , and F. Zhao , “A Critical Review of Microelectrode Arrays and Strategies for Improving Neural Interfaces,” Advanced Healthcare Materials 8 (2019): 1900558. 10.1002/adhm.201900558 PMC6786932 31464094

[62] Z. Huang , Y. Wang , and J. Li , “Intracortical Flexible Microneedle Neural Electrode (f‐µNeurode) Based on Projection‐Micro‐Stereolithography (PµSL) Technology for Chronic in‐Vivo Electrophysiological Recording,” in 2025 IEEE 38th International Conference on Micro Electro Mechanical Systems (MEMS) (IEEE, 2025), 473–476, https://ieeexplore.ieee.org/abstract/document/10917939.

[63] D. Yan , A. Jiman , and D. Ratze , “Microneedle Penetrating Array with Axon‐Sized Dimensions for Cuff‐less Peripheral Nerve Interfacing,” in 2019 9th International IEEE/EMBS Conference on Neural Engineering (NER) (IEEE, 2019), 827–830, https://ieeexplore.ieee.org/document/8717097. PMC12821335 41573096

[64] A. E. Rochford , A. Carnicer‐Lombarte , V. F. Curto , G. G. Malliaras , and D. G. Barone , “When Bio Meets Technology: Biohybrid Neural Interfaces,” Advanced Materials 32 (2020): 1903182. 10.1002/adma.201903182 31517403

[65] A. E. Rochford , A. Carnicer‐Lombarte , M. Kawan , et al., “Functional Neurological Restoration of Amputated Peripheral Nerve Using Biohybrid Regenerative Bioelectronics,” Science Advances 9 (2023): add8162. 10.1126/sciadv.add8162 PMC10032597 36947608

[66] J. J. Shih , D. J. Krusienski , and J. R. Wolpaw , “Brain‐Computer Interfaces in Medicine,” Mayo Clinic Proceedings 87 (2012): 268. 22325364 10.1016/j.mayocp.2011.12.008 PMC3497935

[67] S. K. Mudgal , S. K. Sharma , J. Chaturvedi , and A. Sharma , “Brain Computer Interface Advancement in Neurosciences: Applications and Issues,” Interdisciplinary Neurosurgery 20 (2020): 100694.

[68] C. O'Connor , I. Woods , S. F. McComish , et al., “Biomimetic Scaffolds Enhance iPSC Astrocyte Progenitor Angiogenic, Immunomodulatory, and Neurotrophic Capacity in a Stiffness and Matrix‐Dependent Manner for Spinal Cord Repair Applications,” Advanced Healthcare Materials 14 (2025): 2500830. 40384159 10.1002/adhm.202500830 PMC12184084

[69] Z. Zhao , X. Li , F. He , X. Wei , S. Lin , and C. Xie , “Parallel, Minimally‐invasive Implantation of Ultra‐flexible Neural Electrode Arrays,” Journal of Neural Engineering 16 (2019): 035001. 30736013 10.1088/1741-2552/ab05b6 PMC6506360

[70] N. A. Steinmetz , C. Aydin , A. Lebedeva , et al., “Neuropixels 2.0: A miniaturized high‐density probe for stable, long‐term brain recordings,”. Science 372 (2021): eabf4588. 33859006 10.1126/science.abf4588 PMC8244810

[71] C. Horváth , K. Csikós , B. Árkossy , et al., “Polymer‐based Laminar Probes with an Ultra‐long Flexible Spiral‐shaped Cable for in Vivo Neural Recordings,” Sensors and Actuators B: Chemical 417 (2024): 136220.

[72] E. Musk and Neuralink , “An Integrated Brain‐Machine Interface Platform with Thousands of Channels,” Journal of Medical Internet Research 21 (2019): 16194. 10.2196/16194 PMC6914248 31642810

[73] H. Zhu , “Implanted Electrodes and Microneedle Array Electrodes for the Neuromodulation,” Highlights in Science, Engineering and Technology 23 (2022): 192–197.

[74] J. Li , Y. Ma , D. Huang , et al., “High‐Performance Flexible Microneedle Array as a Low‐Impedance Surface Biopotential Dry Electrode for Wearable Electrophysiological Recording and Polysomnography,” Nano‐Micro Letters 14 (2022): 1–22. 10.1007/s40820-022-00870-0 PMC9198145 35699782

[75] X. Wang , W. Qiu , C. Lu , et al., “Fabrication of Flexible and Conductive Microneedle Array Electrodes from Silk Fibroin by Mesoscopic Engineering,” Advanced Functional Materials 34 (2024): 2311535.

[76] G. Schiavone , X. Kang , F. Fallegger , J. Gandar , G. Courtine , and S. P. Lacour , “Guidelines to Study and Develop Soft Electrode Systems for Neural Stimulation,” Neuron 108 (2020): 238–258. 33120021 10.1016/j.neuron.2020.10.010

[77] T. Hyakumura , U. Aregueta‐Robles , W. Duan , et al., “Improving Deep Brain Stimulation Electrode Performance in Vivo through Use of Conductive Hydrogel Coatings,” Frontiers in neuroscience 15 (2021): 761525. 34803592 10.3389/fnins.2021.761525 PMC8602793

[78] M. E. Uddin , T. Kuila , G. C. Nayak , N. H. Kim , B.‐C. Ku , and J. H. Lee , “Effects of Various Surfactants on the Dispersion Stability and Electrical Conductivity of Surface Modified Graphene,” Journal of Alloys and Compounds 562 (2013): 134–142.

[79] Y. Z. N. Htwe and M. Mariatti , “Surfactant‐assisted Water‐based Graphene Conductive Inks for Flexible Electronic Applications,” Journal of the Taiwan Institute of Chemical Engineers 125 (2021): 402–412.

[80] E. Manousiouthakis , J. Park , J. G. Hardy , J. Y. Lee , and C. E. Schmidt , “Towards the Translation of Electroconductive Organic Materials for Regeneration of Neural Tissues,” Acta Biomaterialia 139 (2022): 22–42. 34339871 10.1016/j.actbio.2021.07.065

[81] J. H. Northrop , “Comparative Hydrolysis of Gelatin by Pepsin, Trypsin, Acid, and Alkali,” Journal of General Physiology 4 (1921): 57–71. 19871916 10.1085/jgp.4.1.57 PMC2140434

[82] A. Griffin , K. Nisi , J. Pepper , et al., “Effect of Surfactant Choice and Concentration on the Dimensions and Yield of Liquid‐Phase‐Exfoliated Nanosheets,” Chemistry of Materials 32 (2020): 2852–2862.

[83] P. May , U. Khan , J. M. Hughes , and J. N. Coleman , “Role of Solubility Parameters in Understanding the Steric Stabilization of Exfoliated Two‐Dimensional Nanosheets by Adsorbed Polymers,” The Journal of Physical Chemistry C 116 (2012): 11393–11400.

[84] T. R. Kyriakides , A. Raj , T. H. Tseng , et al., “Biocompatibility of Nanomaterials and Their Immunological Properties,” Biomed Mater 16 (2021): 10. 10.1088/1748-605X/abe5fa PMC8357854 33578402

[85] J. Mao , R. Guo , and L.‐T. Yan , “Simulation and Analysis of Cellular Internalization Pathways and Membrane Perturbation for Graphene Nanosheets,” Biomaterials 35 (2014): 6069–6077. 24780168 10.1016/j.biomaterials.2014.03.087

[86] J. S. Suk , Q. Xu , N. Kim , J. Hanes , and L. M. Ensign , “PEGylation as a Strategy for Improving Nanoparticle‐based Drug and Gene Delivery,” Advanced Drug Delivery Reviews 99 (2016): 28–51. 26456916 10.1016/j.addr.2015.09.012 PMC4798869

[87] Z. Liu , C. Davis , W. Cai , L. He , X. Chen , and H. Dai , “Circulation and Long‐term Fate of Functionalized, Biocompatible Single‐walled Carbon Nanotubes in Mice Probed by Raman Spectroscopy,” Proceedings of the National Academy of Sciences 105 (2008): 1410–1415. 10.1073/pnas.0707654105 PMC2234157 18230737

[88] R. Bahadur , B. Singh , D. Rai , and R. Srivastava , “Influence of PEGylation on WS _2_ Nanosheets and Its Application in Photothermal Therapy,” ACS Applied Bio Materials 6 (2023): 4740–4748. 10.1021/acsabm.3c00506 37897438

[89] F. M. Veronese and A. Mero , “The Impact of PEGylation on Biological Therapies,” Biodrugs 22 (2008): 315–329. 18778113 10.2165/00063030-200822050-00004

[90] Z. Çiplak , N. Karabudak Yildiz , and A. Çalimli , “Investigation of Graphene/Ag Nanocomposites Synthesis Parameters for Two Different Synthesis Methods,” Fullerenes, Nanotubes and Carbon Nanostructures 23 (2014): 361–370.

[91] N. M. S. Hidayah , W.‐W. Liu , C.‐W. Lai , et al., “Comparison on Graphite, Graphene Oxide and Reduced Graphene Oxide: Synthesis and Characterization,” AIP Conference Proceedings 1892 (2017): 150002, https://pubs.aip.org/aip/acp/article/965987.

[92] X. Wang and Y. Cao , “Characterizations of Absorption, Scattering, and Transmission of Typical Nanoparticles and Their Suspensions,” Journal of Industrial and Engineering Chemistry 82 (2020): 324–332.

[93] T. V. Duncan , “Release of Engineered Nanomaterials from Polymer Nanocomposites: the Effect of Matrix Degradation,” ACS Applied Materials & Interfaces 7 (2015): 20–39. 25397693 10.1021/am5062757

[94] K. Ragaert , L. Cardon , I. D. Baere , and J. Degrieck , Bulk Mechanical Properties of Thermoplastic Poly‐ε‐caprolactone (Ghent University, 2014), 339–344.

[95] H. Ramaraju , A. S. Verga , B. J. Steedley , A. P. Kowblansky , G. E. Green , and S. J. Hollister , “Investigation of the Biodegradation Kinetics and Associated Mechanical Properties of 3D‐printed Polycaprolactone during Long‐term Preclinical Testing,” Biomaterials 321 (2025): 123257. 40154121 10.1016/j.biomaterials.2025.123257 PMC12814369

[96] J. R. Dias , A. Sousa , A. Augusto , P. J. Bártolo , and P. L. Granja , “Electrospun Polycaprolactone (PCL) Degradation: an in Vitro and in Vivo Study,” Polymers 14 (2022): 3397. 36015652 10.3390/polym14163397 PMC9415937

[97] S. Eshraghi and S. Das , “Mechanical and Microstructural Properties of Polycaprolactone Scaffolds with One‐Dimensional, Two‐Dimensional, and Three‐Dimensional Orthogonally Oriented Porous Architectures Produced by Selective Laser Sintering,” Acta Biomaterialia 6 (2010): 2467–2476. 20144914 10.1016/j.actbio.2010.02.002 PMC2874084

[98] J. Noel , “Review of the Properties of Gold Material for MEMS Membrane Applications,” IET Circuits, Devices & Systems 10 (2016): 156–161.

[99] W. D. Callister and D. G. Rethwisch , Materials Science and Engineering: An Introduction: SI Version, (Wiley, 2020).

[100] M. A. Hopcroft , W. D. Nix , and T. W. Kenny , “What Is the Young's Modulus of Silicon?,” Journal of Microelectromechanical Systems 19 (2010): 229–238.

[101] B. Yi , Q. Xu , and W. Liu , “An Overview of Substrate Stiffness Guided Cellular Response and Its Applications in Tissue Regeneration,” Bioact Mater 15 (2021): 82–102. 35386347 10.1016/j.bioactmat.2021.12.005 PMC8940767

[102] D. Stauffer and A. Aharony , Introduction to Percolation Theory, (Taylor & Francis, 2017).

[103] T. Khan , M. S. Irfan , M. Ali , Y. Dong , S. Ramakrishna , and R. Umer , “Insights to Low Electrical Percolation Thresholds of Carbon‐based Polypropylene Nanocomposites,” Carbon 176 (2021): 602–631.

[104] M. Meloni , M. J. Large , J. Miguel , et al., “Explosive Percolation Yields Highly‐conductive Polymer Nanocomposites,” Nature Communications 13 (2022): 1–9. 10.1038/s41467-022-34631-9 PMC9652282 36369509

[105] H.‐J. Choi , M. S. Kim , D. Ahn , S. Y. Yeo , and S. Lee , “Electrical Percolation Threshold of Carbon Black in a Polymer Matrix and Its Application to Antistatic Fibre,” Scientific Reports 9 (2019): 6338. 31004091 10.1038/s41598-019-42495-1 PMC6474880

[106] A. Zareidoost , M. Yousefpour , B. Ghaseme , and A. Amanzadeh , “The Relationship of Surface Roughness and Cell Response of Chemical Surface Modification of Titanium,” Journal of Materials Science: Materials in Medicine 23 (2012): 1479–1488. 22460230 10.1007/s10856-012-4611-9 PMC3368253

[107] V. Brunetti , G. Maiorano , L. Rizzello , et al., “Neurons Sense Nanoscale Roughness with Nanometer Sensitivity,” Proceedings of the National Academy of Sciences 107 (2010): 6264–6269. 10.1073/pnas.0914456107 PMC2851967 20308580

[108] E. Biazar , M. Heidari , A. Asefnejad , and N. Montazeri , “The Relationship between Cellular Adhesion and Surface Roughness in Polystyrene Modified by Microwave Plasma Radiation,” International Journal of Nanomedicine 6 (2011): 631–639. 21698084 10.2147/IJN.S17218 PMC3118690

[109] P. Zhu and Y. Zhao , “Effects of Electrochemical Reaction and Surface Morphology on Electroactive Surface Area of Porous Copper Manufactured by Lost Carbonate Sintering,” RSC Advances 7 (2017): 26392–26400.

[110] Y. Xia , M. Yoshio , and H. Noguchi , “Improved Electrochemical Performance of LiFePO_4_ by Increasing Its Specific Surface Area,” Electrochimica Acta 52 (2006): 240–245.

[111] S. Kumar , S. Bose , and K. Chatterjee , “Amine‐functionalized Multiwall Carbon Nanotubes Impart Osteoinductive and Bactericidal Properties in Poly(ε‐caprolactone) Composites,” RSC Advances 4 (2014): 19086–19098.

[112] S. M. Wellman , J. R. Eles , K. A. Ludwig , et al., “A Materials Roadmap to Functional Neural Interface Design,” Advanced Functional Materials 28 (2018): 1701269. 29805350 10.1002/adfm.201701269 PMC5963731

[113] M. Gori , G. Vadalà , S. M. Giannitelli , V. Denaro , and G. Di Pino , “Biomedical and Tissue Engineering Strategies to Control Foreign Body Reaction to Invasive Neural Electrodes,” Front Bioeng Biotechnol 9 (2021): 659033. 34113605 10.3389/fbioe.2021.659033 PMC8185207

[114] K. T. Baldwin , K. K. Murai , and B. S. Khakh , “Astrocyte Morphology,” Trends in Cell Biology 34 (2024): 547–565. 38180380 10.1016/j.tcb.2023.09.006 PMC11590062

[115] C. Boehler , S. Carli , L. Fadiga , T. Stieglitz , and M. Asplund , “Tutorial: Guidelines for Standardized Performance Tests for Electrodes Intended for Neural Interfaces and Bioelectronics,” Nature Protocols 15 (2020): 3557–3578. 33077918 10.1038/s41596-020-0389-2

[116] H. Tomiyasu , H. Shikata , K. Takao , N. Asanuma , S. Taruta , and Y.‐Y. Park , “An Aqueous Electrolyte of the Widest Potential Window and Its Superior Capability for Capacitors,” Scientific Reports 7 (2017): 45048. 28322349 10.1038/srep45048 PMC5359556

[117] S. J. Wilks , S. M. Richardson‐Burn , J. L. Hendricks , D. Martin , and K. J. Otto , “Poly(3,4‐ethylene dioxythiophene) (PEDOT) as a Micro‐neural Interface Material for Electrostimulation,” Frontiers in Neuroengineering 2 (2009): 1–8, https://www.frontiersin.org/journals/neuroengineering/articles/10.3389/neuro.16.007.2009/full. 19543541 10.3389/neuro.16.007.2009 PMC2697029

[118] Q. Zeng , S. Yu , Z. Fan , Y. Huang , B. Song , and T. Zhou , “Nanocone‐Array‐Based Platinum‐Iridium Oxide Neural Microelectrodes: Structure, Electrochemistry, Durability and Biocompatibility Study,” Nanomaterials 12 (2022): 3445. 36234573 10.3390/nano12193445 PMC9565584

[119] D. W. Kumsa , N. Bhadra , E. M. Hudak , S. C. Kelley , D. F. Untereker , and J. T. Mortimer , “Electron Transfer Processes Occurring on Platinum Neural Stimulating Electrodes: a Tutorial on the i(Ve) Profile,” Journal of Neural Engineering 13 (2016): 052001. 27518125 10.1088/1741-2560/13/5/052001

[120] C. M. Lewis , C. Boehler , R. Liljemalm , P. Fries , T. Stieglitz , and M. Asplund , “Recording Quality Is Systematically Related to Electrode Impedance,” Advanced Healthcare Materials 13 (2024): 2303401. 10.1002/adhm.202303401 38354063

[121] D. Gupta , A. K. Singh , N. Kar , A. Dravid , and J. Bellare , “Modelling and Optimization of NaOH‐etched 3‐D Printed PCL for Enhanced Cellular Attachment and Growth with Minimal Loss of Mechanical Strength,” Materials Science and Engineering: C 98 (2019): 602–611. 30813063 10.1016/j.msec.2018.12.084

[122] M. Schneider , N. Fritzsche , A. Puciul‐Malinowska , et al., “Surface Etching of 3D Printed Poly(lactic acid) with NaOH: a Systematic Approach,” Polymers 12 (2020): 1711. 32751597 10.3390/polym12081711 PMC7464172

[123] Z.‐X. Zhou , Y.‐R. Chen , J.‐Y. Zhang , et al., “Facile Strategy on Hydrophilic Modification of Poly(ε‐caprolactone) Scaffolds for Assisting Tissue‐Engineered Meniscus Constructs in Vitro,” Front Pharmacol 11 (2020): 471. 32431606 10.3389/fphar.2020.00471 PMC7216581

[124] M. Dupont , A. F. Hollenkamp , and S. W. Donne , “Electrochemically Active Surface Area Effects on the Performance of Manganese Dioxide for Electrochemical Capacitor Applications,” Electrochimica Acta 104 (2013): 140–147.

[125] R. Heimböckel , F. Hoffmann , and M. Fröba , “Insights into the Influence of the Pore Size and Surface Area of Activated Carbons on the Energy Storage of Electric Double Layer Capacitors with a New Potentially Universally Applicable Capacitor Model,” Physical Chemistry Chemical Physics 21 (2019): 3122–3133. 30675602 10.1039/c8cp06443a

[126] G. Höflinger , Brief Introduction to Coating Technology for Electron Microscopy (2013). https://www.leica‐microsystems.com/science‐lab/life‐science/brief‐introduction‐to‐coating‐technology‐for‐electron‐microscopy/.

[127] A. R. Harris , C. Newbold , P. Carter , R. Cowan , and G. G. Wallace , “Measuring the Effective Area and Charge Density of Platinum Electrodes for Bionic Devices,” Journal of Neural Engineering 15 (2018): 046015. 29595147 10.1088/1741-2552/aaba8b

[128] G. Dijk , H. J. Ruigrok , and R. P. O'Connor , “PEDOT:PSS‐Coated Stimulation Electrodes Attenuate Irreversible Electrochemical Events and Reduce Cell Electropermeabilization,” Advanced Materials Interfaces 8 (2021): 2100214.

[129] A. Cisnal , J. C. Fraile , J. Pérez‐Turiel , V. Muñoz‐Martinez , C. Müller , and F. R. Ihmig , “A Measurement Setup and Automated Calculation Method to Determine the Charge Injection Capacity of Implantable Microelectrodes,” Sensors (Basel, Switzerland) 18 (2018): 4152. 30486353 10.3390/s18124152 PMC6308657

[130] M. Ganji , A. Tanaka , V. Gilja , E. Halgren , and S. A. Dayeh , “Scaling Effects on the Electrochemical Stimulation Performance of Au, Pt, and PEDOT:PSS Electrocorticography Arrays,” Advanced Functional Materials 27 (2017): 1703019.

[131] J. D. Weiland , D. J. Anderson , and M. S. Humayun , “In Vitro Electrical Properties for Iridium Oxide versus Titanium Nitride Stimulating Electrodes,” IEEE Transactions on Biomedical Engineering 49 (2002): 1574–1579. 12549739 10.1109/TBME.2002.805487

[132] K. T. M. Tran and T. D. Nguyen , “Lithography‐based Methods to Manufacture Biomaterials at Small Scales,” Journal of Science: Advanced Materials and Devices 2 (2017): 1–14.

[133] E. M. Maynard , C. T. Nordhausen , and R. A. Normann , “The Utah Intracortical Electrode Array: a Recording Structure for Potential Brain‐computer Interfaces,” Electroencephalography and Clinical Neurophysiology 102 (1997): 228–239. 9129578 10.1016/s0013-4694(96)95176-0

[134] J. Kaur , L. M. Fahmy , E. Davoodi‐Bojd , et al., “Waste Clearance in the Brain,” Frontiers in Neuroanatomy 15 (2021): 665803. 34305538 10.3389/fnana.2021.665803 PMC8292771

[135] Y. Cheng and J. Haorah , “How Does the Brain Remove Its Waste Metabolites from Within?,” International Journal of Physiology, Pathophysiology and Pharmacology 11 (2019): 238–249. 31993098 PMC6971497

[136] N. H. Hosseini , R. Hoffmann , S. Kisban , T. Stieglitz , O. Paul , and P. Ruther , “Comparative Study on the Insertion Behavior of Cerebral Microprobes,” in 2007 29th Annual International Conference of the IEEE Engineering in Medicine and Biology Society , (IEEE, 2007), 4711–4714, http://ieeexplore.ieee.org/document/4353391. 10.1109/IEMBS.2007.4353391 18003057

[137] G. Milior , M. A. Di Castro , L. P. Sciarria , et al., “Electrophysiological Properties of CA1 Pyramidal Neurons along the Longitudinal Axis of the Mouse Hippocampus,” Scientific Reports 6 (2016): 38242. 27922053 10.1038/srep38242 PMC5138623

[138] R. Malik , K. A. Dougherty , K. Parikh , C. Byrne , and D. Johnston , “Mapping the Electrophysiological and Morphological Properties of CA1 Pyramidal Neurons along the Longitudinal Hippocampal Axis,” Hippocampus 26 (2016): 341–361. 26333017 10.1002/hipo.22526 PMC4760884

[139] B. N. Routh , D. Johnston , K. Harris , and R. A. Chitwood , “Anatomical and Electrophysiological Comparison of CA1 Pyramidal Neurons of the Rat and Mouse,” Journal of Neurophysiology 102 (2009): 2288–2302. 19675296 10.1152/jn.00082.2009 PMC2775381

[140] P. Oldroyd , J. Gurke , and G. G. Malliaras , “Stability of Thin Film Neuromodulation Electrodes under Accelerated Aging Conditions,” Advanced Functional Materials 33 (2022): 2208881.

[141] J. Schulte , D. Ashouri , and T. Stieglitz , “The Longevity of Neural Interfaces—Mechanical Oscillation of Thin Film Metal‐Based Neural Electrodes Determine Stability during Electrical Stimulation,” Advanced Functional Materials 34 (2023): 2310130.

[142] S. Lv , Z. Xu , F. Mo , et al., “Long‐term Stability Strategies of Deep Brain Flexible Neural Interface,” npj Flex Electron 9 (2025): 1–17.

[143] M. Xia , B. N. Agca , T. Yoshida , et al., “Scalable, Flexible Carbon fiber Electrode Thread Arrays for Three‐Dimensional Probing of Neurochemical Activity in Deep Brain Structures of Rodents,” Biosensors and Bioelectronics 241 (2023): 115625. 37708685 10.1016/j.bios.2023.115625 PMC10591823

[144] M. D. Ferro , C. M. Proctor , A. Gonzalez , et al., “NeuroRoots, a Bio‐inspired, Seamless Brain Machine Interface for Long‐term Recording in Delicate Brain Regions,” AIP Advances 14 (2024): 085109. 39130131 10.1063/5.0216979 PMC11309783

[145] S. T. Keene , A. Rao , and G. G. Malliaras , “The Relationship between Ionic‐electronic Coupling and Transport in Organic Mixed Conductors,” Science Advances 9 (2023): adi3536. 10.1126/sciadv.adi3536 PMC10468126 37647402

[146] P. Oldroyd , S. E. Hadwe , D. G. Barone , and G. G. Malliaras , “Thin‐film Implants for Bioelectronic Medicine,” MRS Bulletin 49 (2024): 1045–1058, 10.1557/s43577-024-00786-7. 39397879 PMC11469980

[147] M. Kim , H. Lee , S. Nam , D.‐H. Kim , and G. D. Cha , “Soft Bioelectronics Using Nanomaterials and Nanostructures for Neuroengineering,” Accounts of Chemical Research 57 (2024): 1633–1647, 10.1021/acs.accounts.4c00163. 38752397

[148] A. Domínguez‐Bajo , J. M. Rosa , A. González‐Mayorga , et al., “Nanostructured Gold Electrodes Promote Neural Maturation and Network Connectivity,” Biomaterials 279 (2021): 121186. 34700221 10.1016/j.biomaterials.2021.121186

[149] R. Garg , G. Balakrishnan , R. B. Rashid , et al., “Graphene and Poly(3,4‐ethylenedioxythiophene)–Polystyrene Sulfonate Hybrid Nanostructures for Input/Output Bioelectronics,” ACS Applied Nano Materials 6, no. 10 (2023): 8495–8505, 10.1021/ACSANM.3C00849.

[150] C. Backes , B. M. Szydłowska , A. Harvey , et al., “Production of Highly Monolayer Enriched Dispersions of Liquid‐exfoliated Nanosheets by Liquid Cascade Centrifugation,” ACS Nano 10 (2016): 1589–1601. 26728793 10.1021/acsnano.5b07228

[151] D. Nečas and P. Klapetek , “Gwyddion: an Open‐source Software for SPM Data Analysis,” Central European Journal of Physics 10 (2012): 181–188.

[152] P. A. Nistor , P. W. May , F. Tamagnini , A. D. Randall , and M. A. Caldwell , “Long‐term Culture of Pluripotent Stem‐cell‐derived human Neurons on Diamond – A Substrate for Neurodegeneration Research and Therapy,” Biomaterials 61 (2015): 139–149. 26002787 10.1016/j.biomaterials.2015.04.050

[153] S. F. McComish and M. A. Caldwell , “Generation of Defined Neural Populations from Pluripotent Stem Cells,” Philosophical Transactions of the Royal Society B: Biological Sciences 373 (2018): 20170214. 10.1098/rstb.2017.0214 PMC5974438 29786550

[154] L. A. Crompton , S. F. McComish , P. Stathakos , O. Cordero‐Llana , J. D. Lane , and M. A. Caldwell , “Efficient and Scalable Generation of human Ventral Midbrain Astrocytes from human‐induced Pluripotent Stem Cells,” Journal of Visualized Experiments 2 (2021): 1–19. 10.3791/62095 34661566

[155] A. Serio , B. Bilican , S. J. Barmada , et al., “Astrocyte Pathology and the Absence of Non‐cell Autonomy in an Induced Pluripotent Stem Cell Model of TDP‐43 Proteinopathy,” Proceedings of the National Academy of Sciences 110 (2013): 4697–4702. 10.1073/pnas.1300398110 PMC3607024 23401527

[156] E. M. Cahill and E. D. O'Cearbhaill , “Toward Biofunctional Microneedles for Stimulus Responsive Drug Delivery,” Bioconjugate Chemistry 26 (2015): 1289–1296. 26020359 10.1021/acs.bioconjchem.5b00211

[157] K. J. Krieger , N. Bertollo , M. Dangol , J. T. Sheridan , M. M. Lowery , and E. D. O'Cearbhaill , “Simple and Customizable Method for Fabrication of High‐aspect Ratio Microneedle Molds Using Low‐cost 3D Printing,” Microsyst Nanoeng 5 (2019): 1–14. 31645996 10.1038/s41378-019-0088-8 PMC6799892

