# Supplementary material for: PolyGraph – Flexible, Biocompatible & Electrically Optimized Graphene‐Polymer Composites for Next‐Generation Neural Interfaces
Source: Adv Healthc Mater. 2026 May 12;15(23):e05076. doi: 10.1002/adhm.202505076 (PMC13280202; doi:10.1002/adhm.202505076)
Supplement: Supplementary file 1 — Supporting File: adhm71176‐sup‐0001‐SuppMat.docx. [file ADHM-15-0-s001.docx]

# Supporting Information

**PolyGraph – Flexible, Biocompatible & Electrically Optimised Graphene-Polymer Composites for Next-Generation Neural Interfaces**

*Jack Maughan, Ian Woods, Cian O’Connor, Pablo Quintana-Sarti, Eoin Caffrey, Jose M. Munuera, Adrian Dervan, Alejandro López Valdés, Omar Mamad, Maeve A. Caldwell, Fergal J. O’Brien, Jonathan N. Coleman**

*
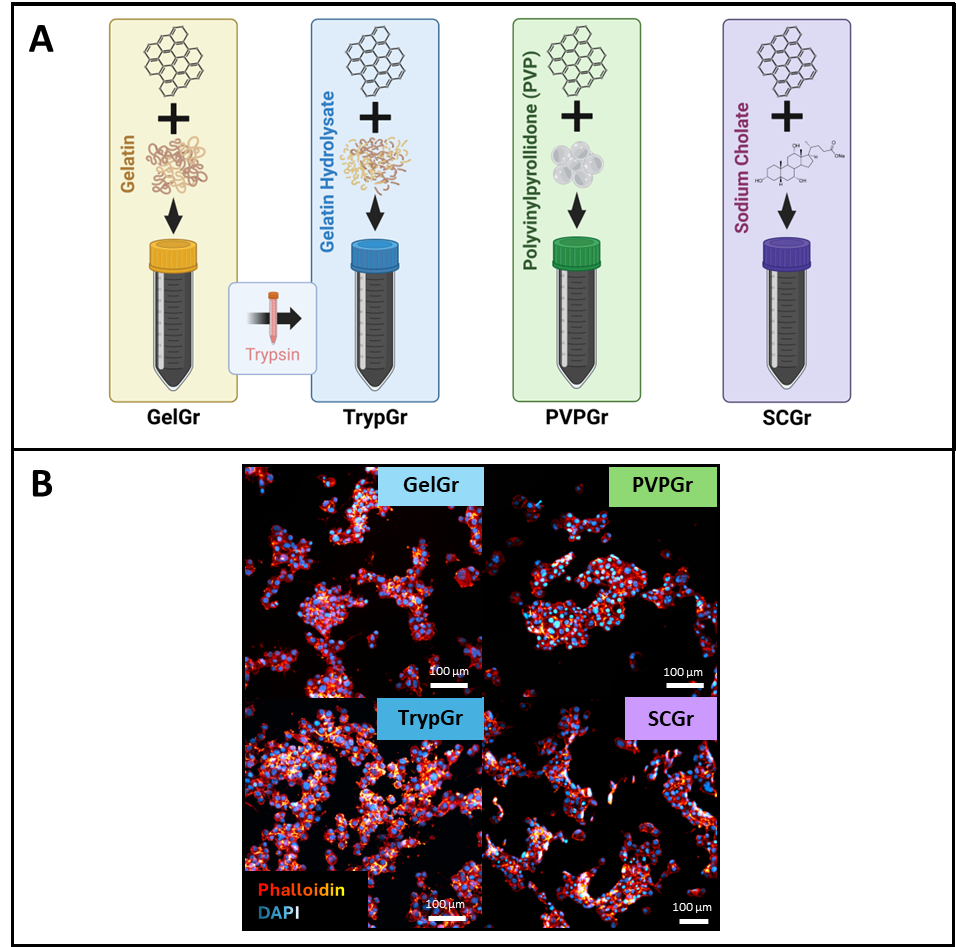
*

***Figure S1. Biological characterisation of graphene candidates: A)*** *Schematic of graphene formulations, highlighting stabilisation molecules used: gelatin (GelGr)*, *gelatin hydrolysate post-trypsin treatment (TrypGr), polyvinylpyrrolidone (PVPGr), and sodium cholate (SCGr).* ***B)*** *Representative immunofluorescence images of cells cultured on collagen-graphene films of each formulation. Phalloidin: Yellow/red, DAPI: Blue/white. Scale bars 100 μm. Significances: *p < 0.05, **p < 0.01, ***p < 0.001, ****p < 0.0001.*

*
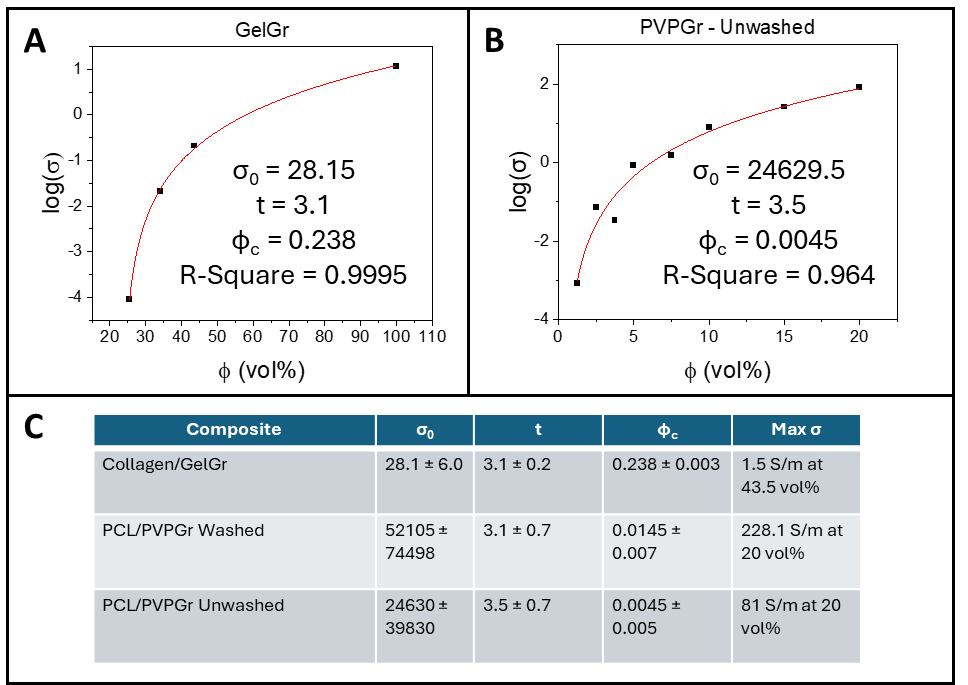
*

***Figure S2. Percolation curves:*** ***A-B)*** *Percolation curves for GelGr (A) and unwashed PVPGr (B).* ***C)*** *Summary of percolation fitting parameters (σ₀, t, ϕ_c_, and maximum σ), highlighting the superior conductivity of PolyGraph versus collagen/GelGr, and the critical role of washing in enhancing electrical properties.*

***
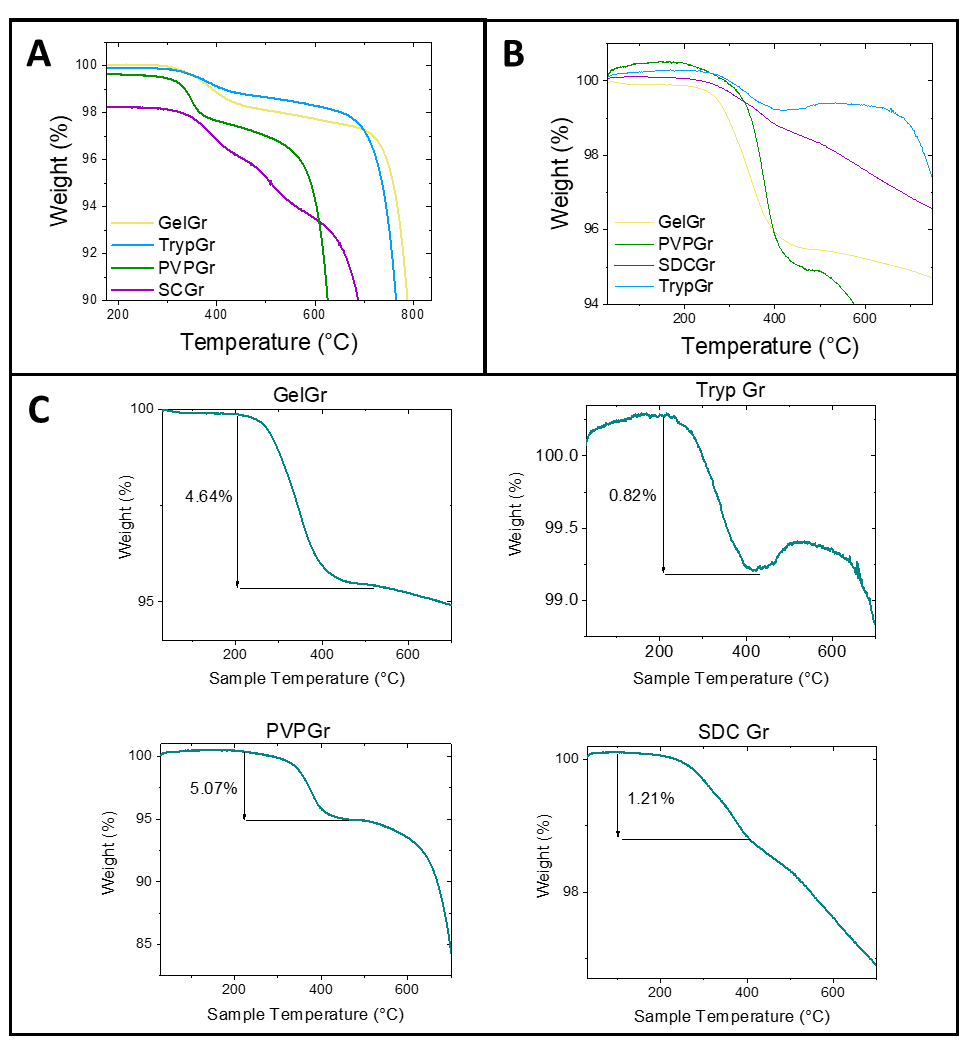
Figure S3. Thermogravimetric analysis (TGA) of graphene candidates:*** ***A)****TGA curves in air, providing estimates of the surface coating thickness for each formulation.* ***B)****TGA performed under nitrogen atmosphere shows similar trends for GelGr, TrypGr, PVPGr, and SCGr.* ***C)*** *Individual TGA traces for each graphene type, highlighting percentage weight loss associated with polymer removal.*

*
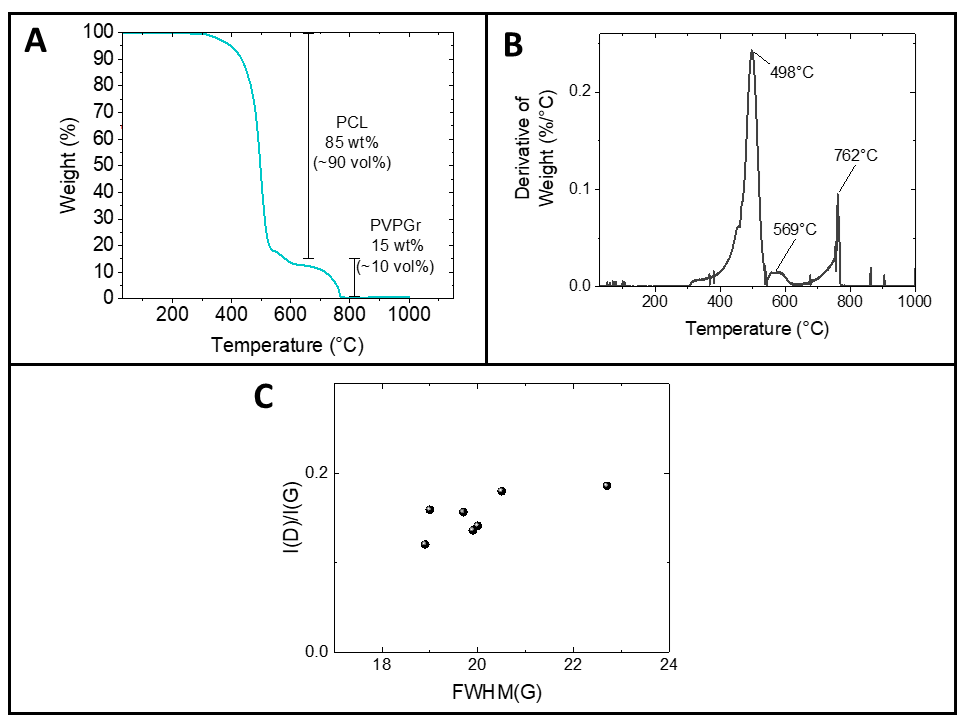
****Figure S4. Further physical characterisation of PVPGr and PolyGraph: A)*** *TGA of PolyGraph, verifying accurate formulation composition.* ***B)*** *Derivative of TGA spectrum for PolyGraph10%, showing decomposition peaks corresponding to PCL (~500 °C) and PVPGr (~750 °C) respectively.* ***C)****Raman analysis of PVPGr, showing no correlation between the I_D_/I_G_ ratio and the full-width-half-maximum (FWHM) of the G peak, indicating a defect-free basal plane.* *Plotting the intensity ratio of the D and G peaks* $\left( \frac{I_{D}}{I_{G}} \right)$ *against the full-width-half-maximum of the G peak (FWHM_G_), revealed no correlation strongly suggesting that the observed D peak and associated disorder are not caused by basal plane imperfections, but rather by edge defects,^158^ consistent with previously reported LPE graphene inks.^159^*^,^*^160^ Building on this, we can estimate the average lateral size of the nanosheets (<L>) using the relationship* $\frac{I_{D}}{I_{G}} \approx\left( \frac{I_{D}}{I_{G}} \right)_{{}^{graphite}}+\frac{k}{<L>}$*, with k = 0.17.^161^ Using* $\frac{I_{D}}{I_{G}}\approx0.05 \left( \pm0.05 \right)$ *for our graphite^162^ and an average* $\frac{I_{D}}{I_{G}}$*of 0.15 for the exfoliated graphene flakes, the lateral size of the nanosheets can be estimated to be 1.13 – 3.4 µm.*

***
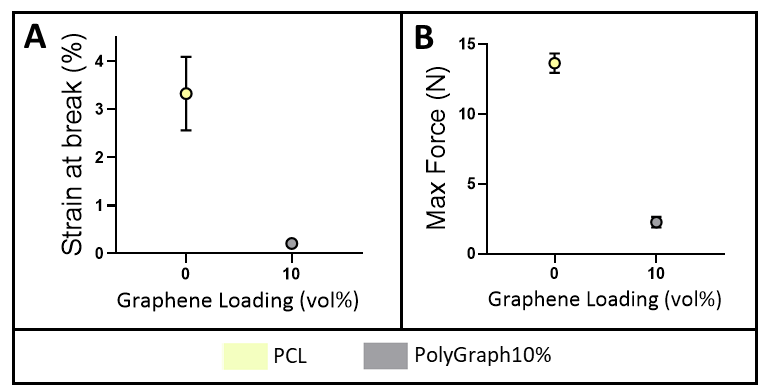
****
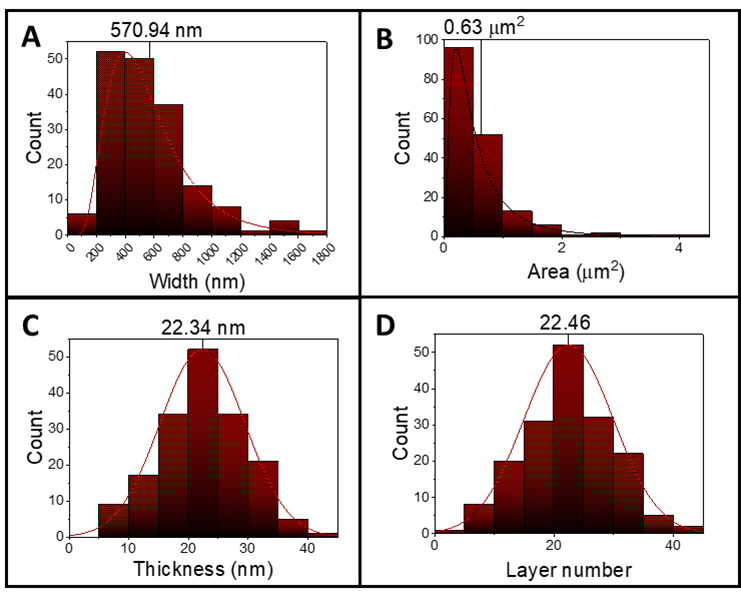
****Figure S5. AFM analysis of PVPGr nanosheets:*** *Histograms of* ***A)*** *nanosheet width* ***B)****nanosheet area* ***C)*** *nanosheet thickness and* ***D)*** *estimated layer number, based on analysis of 173 flakes. Mean values indicated above each plot.*

***Figure S6. Mechanical testing of PolyGraph: A-B)*** *Strain at break (A) and maximum force (B) measurements for PCL and PolyGraph10%, showing reduced ductility following graphene incorporation.*

***
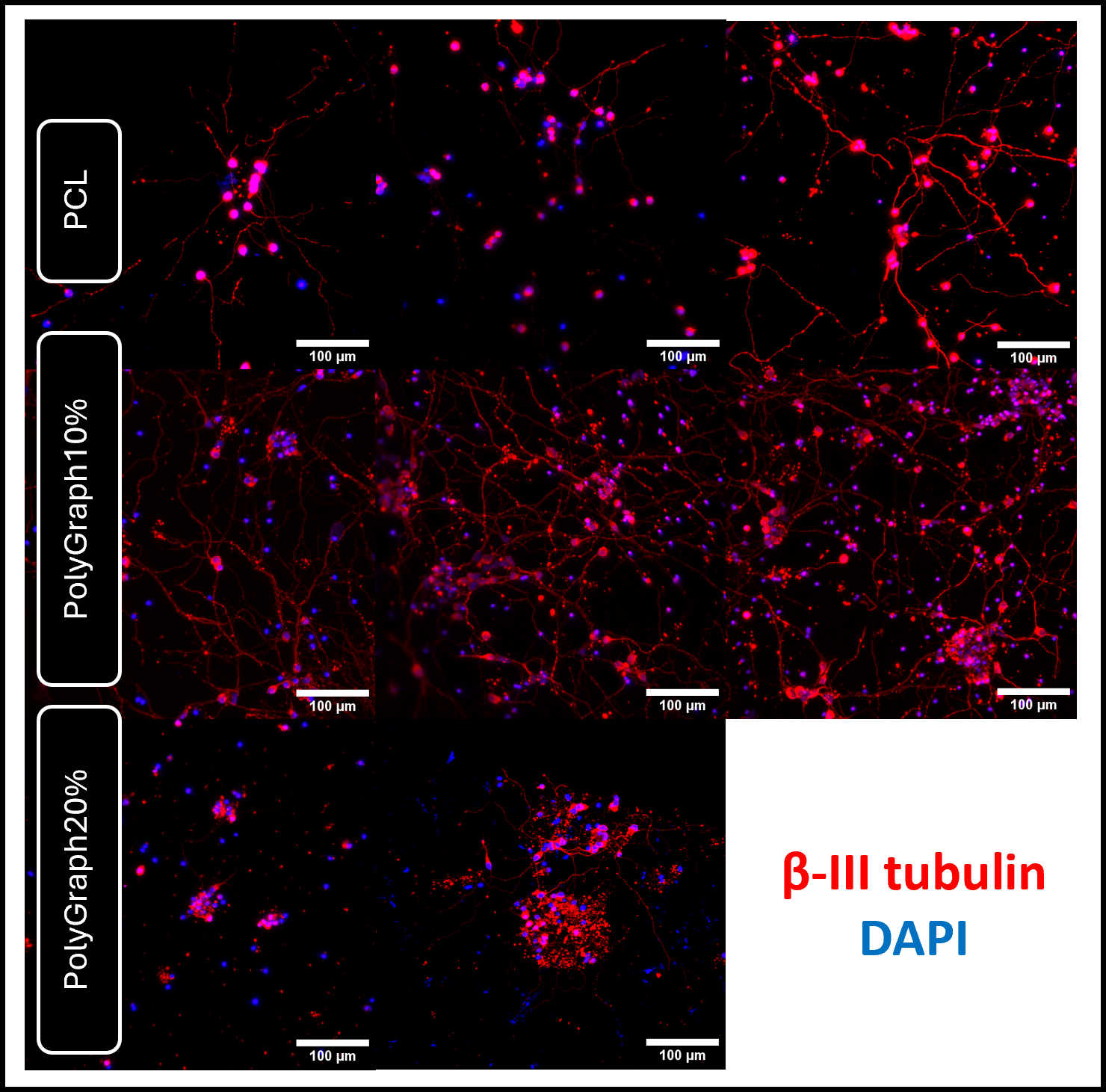
Figure S7. iPSC-derived neurons cultured on PolyGraph:*** *iPSC-derived neurons were cultured on PolyGraph for 14 days and imaged using confocal microscopy. Extensive neurite outgrowth and network formation are visible on PolyGraph10%, indicating its support of neuronal growth. Scale bars 100 µm.*

*
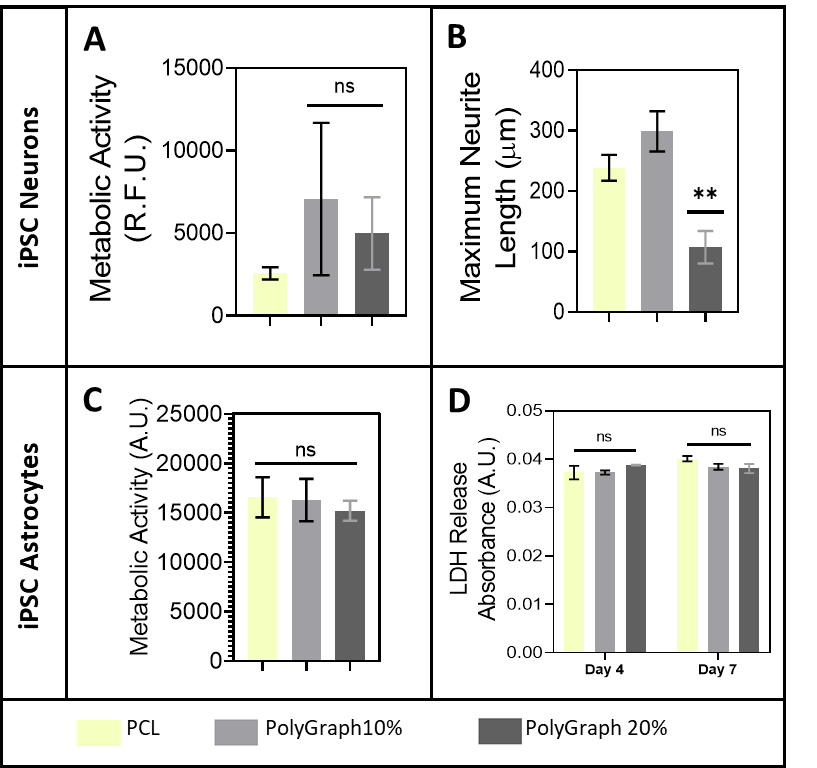
*

***Figure S8. Biological and immunological assessment of PolyGraph:*** ***A)****Metabolic activity of iPSC-derived neurons cultured on PolyGraph.* ***B)*** *Maximum neurite length of iPSC-derived neurons, showing a significant increase on PolyGraph10%. Scale bars 100 μm.* ***C)*** *LDH release from iPSC-derived astrocytes, indicating no significant cytotoxicity on PolyGraph.* ***D)****Metabolic activity of iPSC-derived astrocytes at day 14, showing no significant differences. Significances: **p < 0.01.*

*
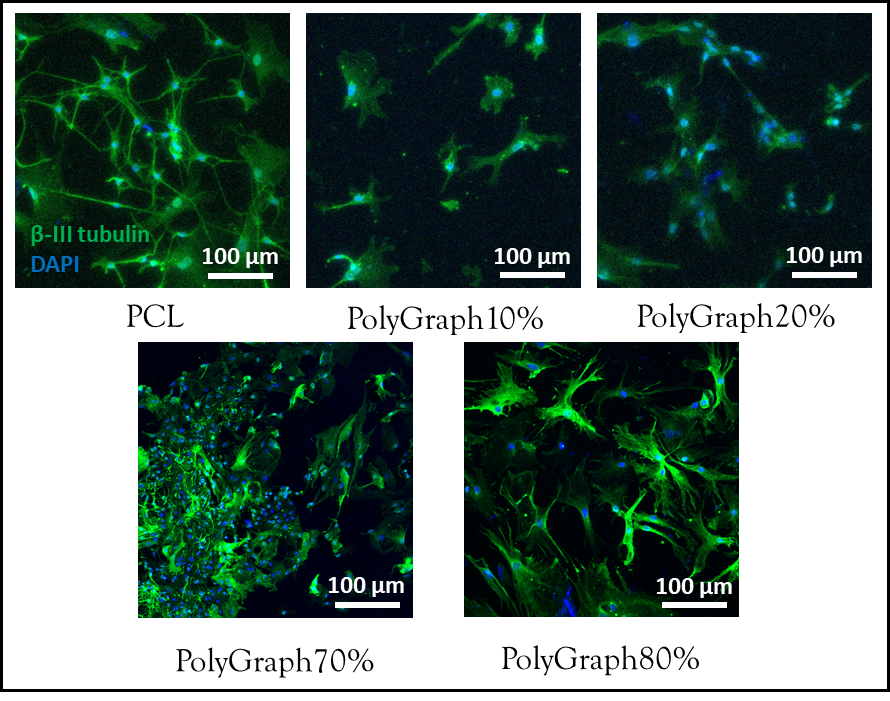
****Figure S9. Mouse primary cortical neurons cultured on PolyGraph:*** *Mouse primary neurons were cultured on PolyGraph composites for 14 days and imaged via confocal microscopy. Robust survival on PolyGraph composites indicates biocompatibility with sensitive, physiologically relevant cells. Scale bars 100 μm.*

***
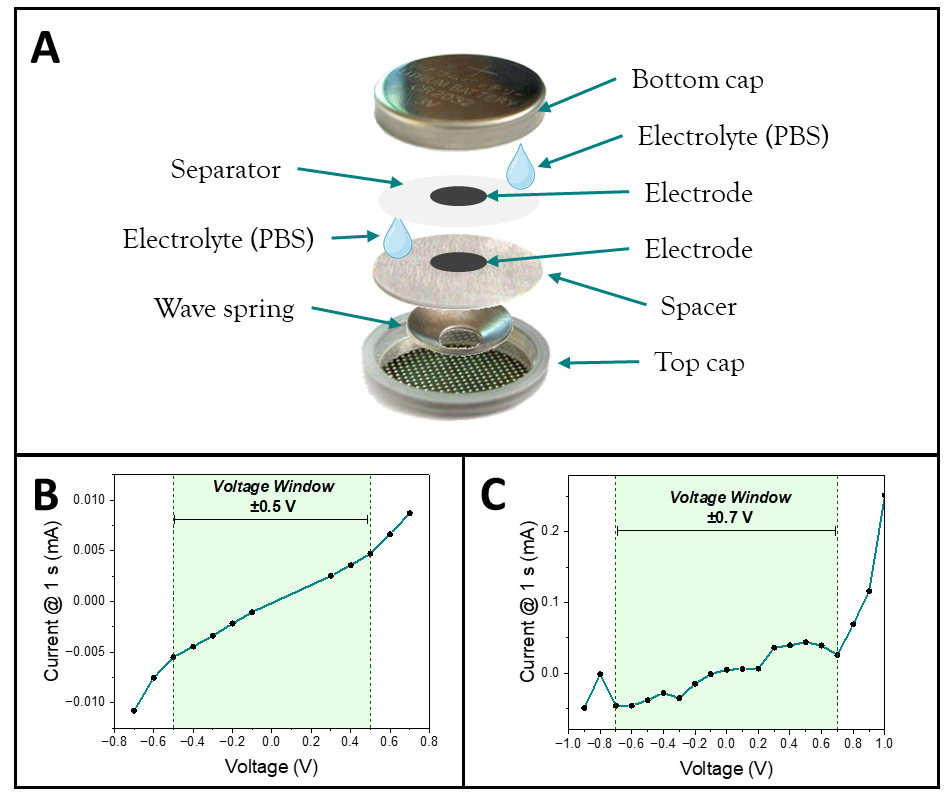
Figure S10. Electrical characterisation of PVPGr and PolyGraph composites:*** ***A)****Schematic of two-electrode coin cell setup used for electrochemical characterisation.* ***B)****Chronoamperometry data showing the potential window of untreated PolyGraph at ±0.5 V.* ***C)*** *Chronoamperometry of AuPd + NaOH-treated PolyGraph, indicating an expanded potential window of approximately ±0.7 V.*

*
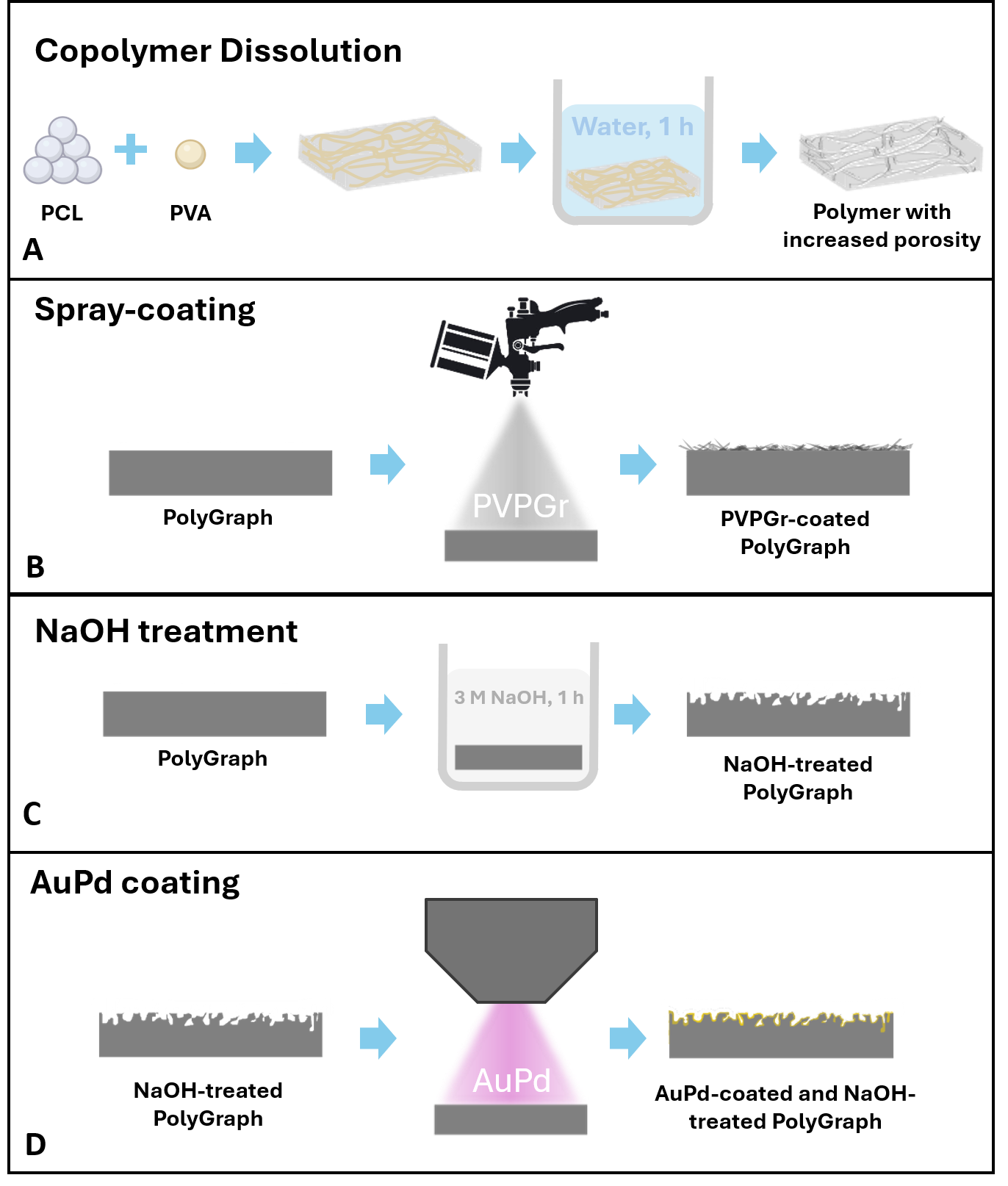
****Figure S11. Techniques for enhancing electrochemical properties of PolyGraph: A)****Copolymer dissolution* ***B)*** *Spray coating with PVPGr* ***C)*** *NaOH treatment of PCL* ***D)*** *AuPd coating of PolyGraph.*

***
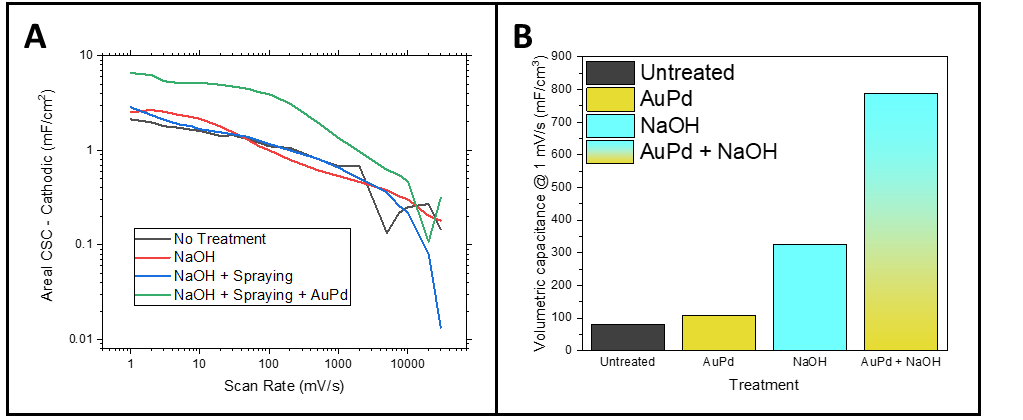
Figure S12. Charge storage capacity enhancement of PolyGraph by surface treatment: A)****Areal charge storage capacity (CSC) of PolyGraph composites as a function of scan rate, showing the effects of NaOH roughening, graphene spray coating, and AuPd sputter coating individually and in combination.* ***B)*** *Volumetric capacitance at 1 mV/s for untreated and treated PolyGraph composites, highlighting substantial improvements with combined NaOH and AuPd treatments.*

***
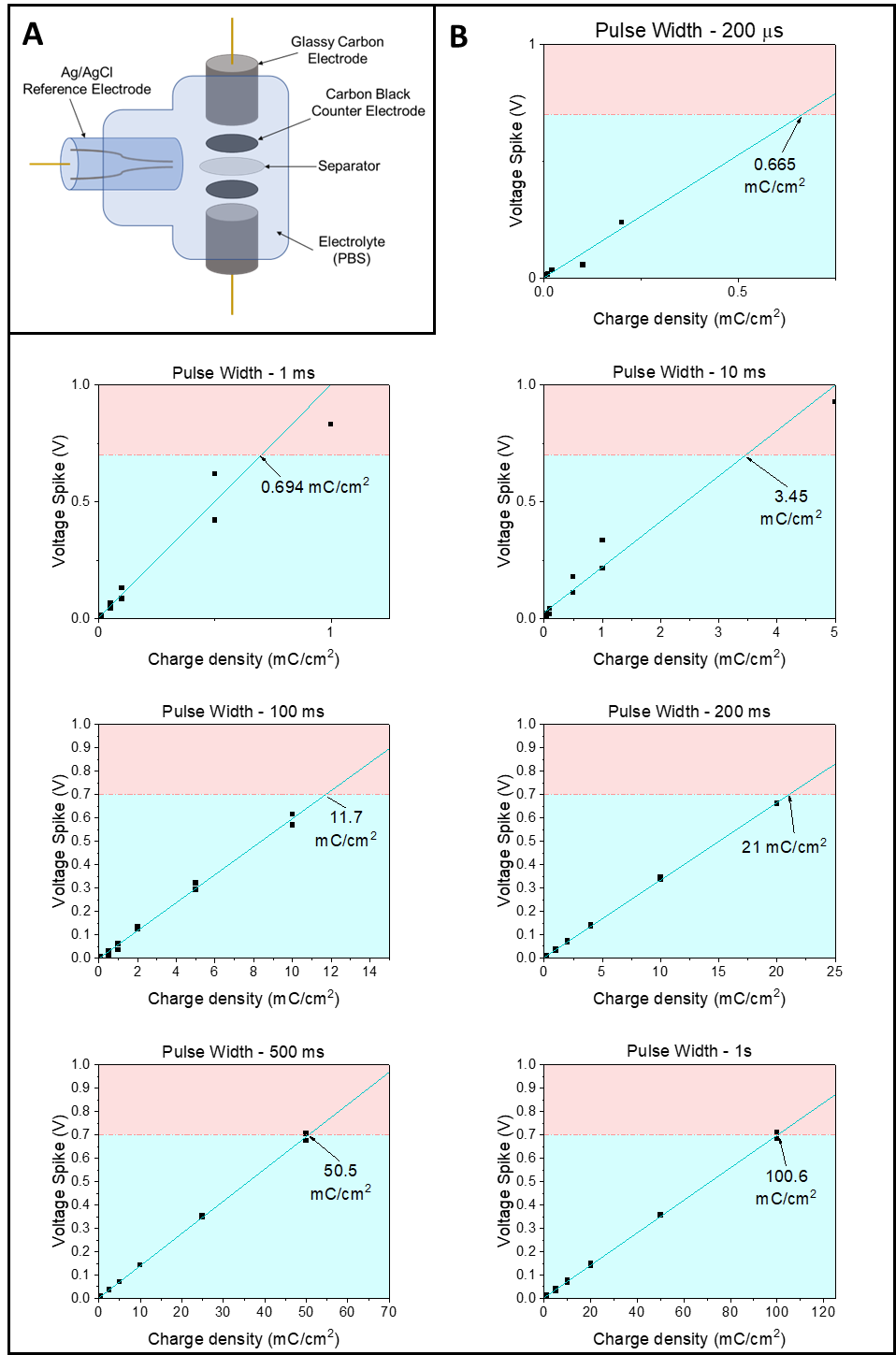
Figure S13. Voltage transients curves:*** ***A)*** *Schematic of three-electrode setup used to capture voltage transients following square wave excitation* ***B)*** *Voltage transient responses at pulse widths from 200 µs to 1 s, used to calculate charge injection capacity (CIC). The electrochemical potential window (~±0.7 V) is shown in blue.*

***
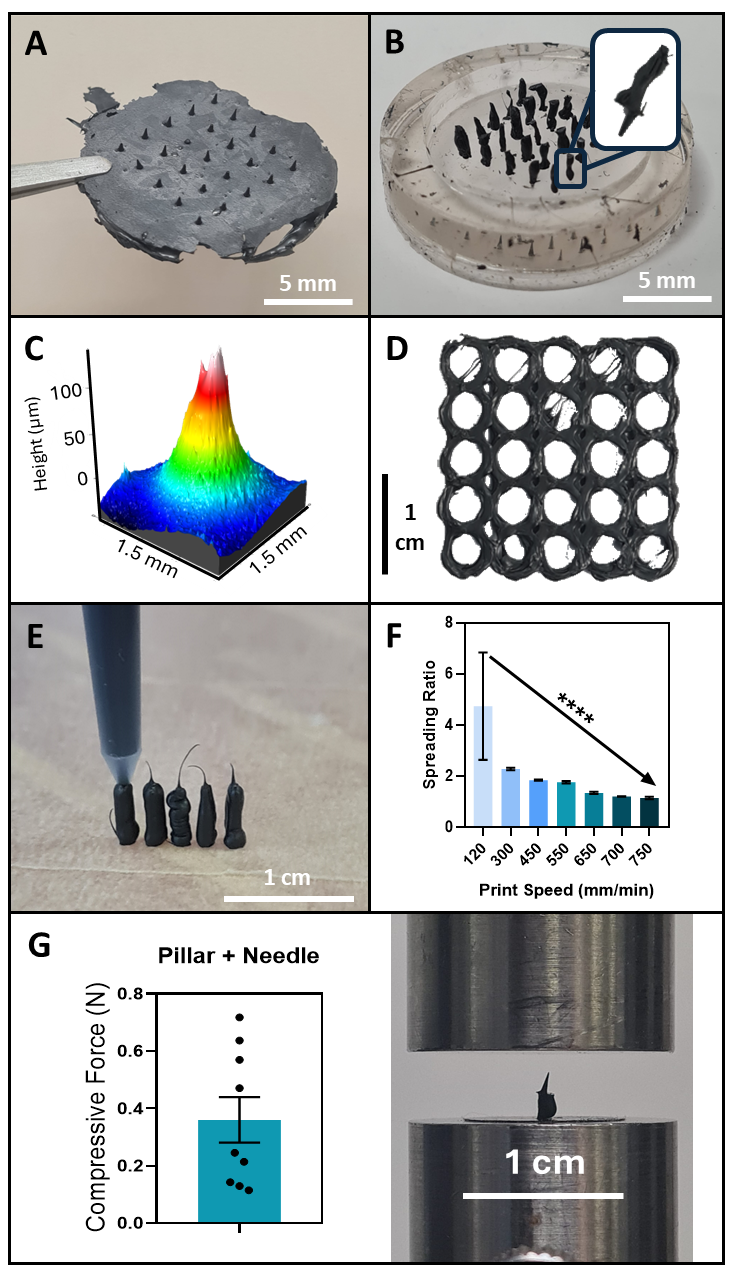
Figure S14. 3D printing of PolyGraph: A)*** *Dry-cast microneedle array.* ***B)*** *Microneedle array with pillar backing, in silicone mold.* ***C)*** *White-light interferometry profile of an individual microneedle.* ***D)*** *Cylindrical scaffold 3D printed from PolyGraph10% for tissue engineering applications.* ***E)*** *Optical images of 3D-printed pillars, enabling microneedle isolation.* ***F)****Determination of optimal printing speed for reduced spreading ratio in solvent 3D printing of PolyGraph.* ***G)*** *Compressive modulus testing of microneedles, assessing robustness for tissue insertion. Scale bars: A-B, 5 mm; D, E, G, 1 cm. Significances: ****p < 0.0001.*

*
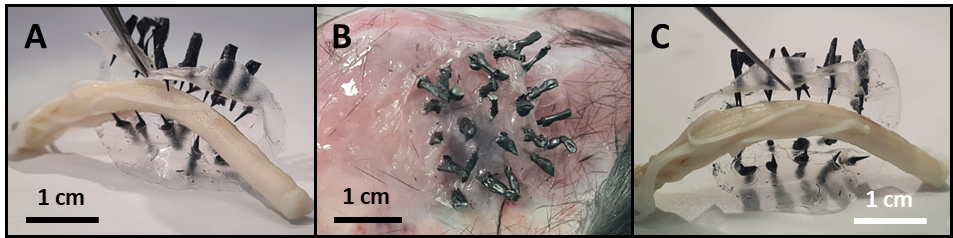
*

***Figure S15. Ex-vivo application of flexible microneedle arrays: A-C)*** *Images of flexible PolyGraph microneedle arrays on ex-vivo samples of embalmed rat spinal cord (A & C), and mouse back tissue (B), demonstrating conformability and penetration. Scale bars 1 cm.*

***
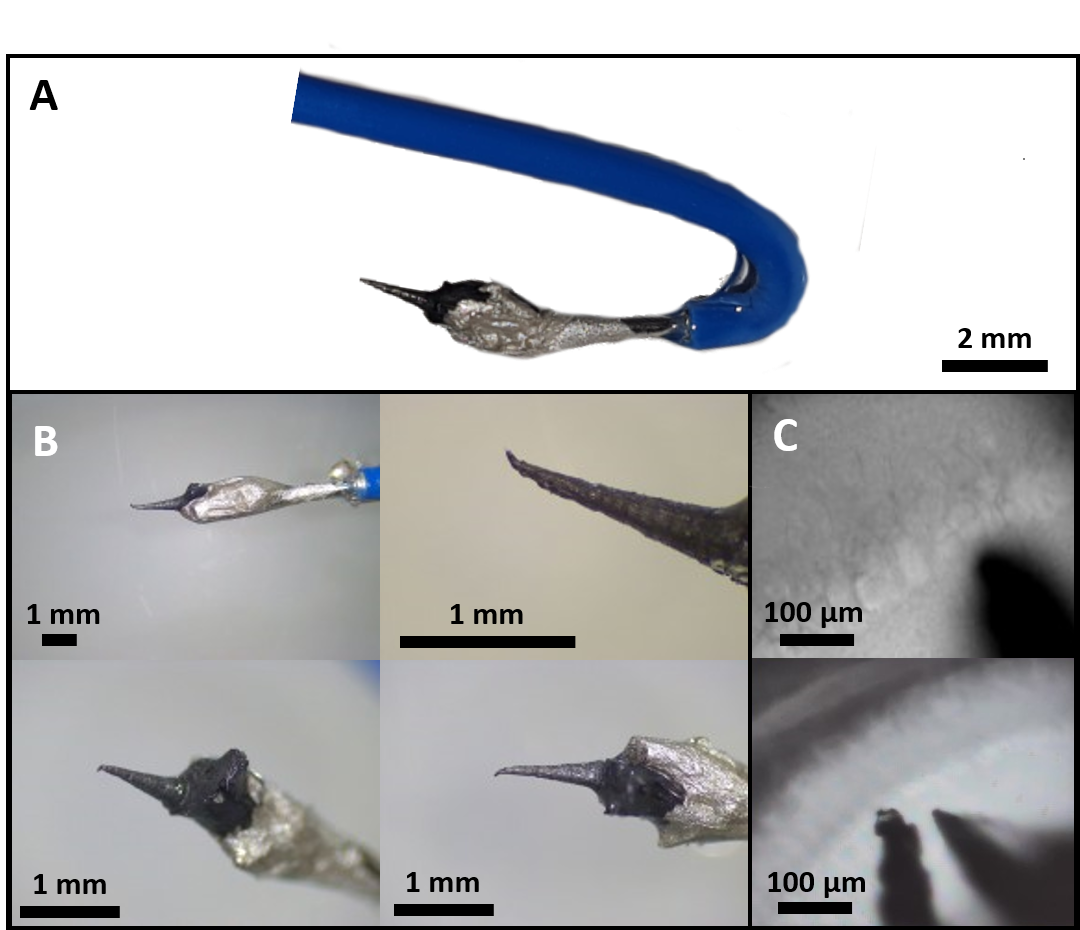
***

***Figure S16. PolyGraph microneedles prepared for electrophysiological measurements: A‑B)****PDMS-sheathed PolyGraph microneedle electrodes following NaOH and AuPd treatments, connected to wiring using silver paint.* ***C)*** *Optical microscope image of microneedles inserted into stratum radiatum of CA1 hippocampal region, showing the sharp microneedle tip surrounded by pyramidal neurons. Scalebars: A, 2 mm; B, 1 mm; C, 100 μm.*

*
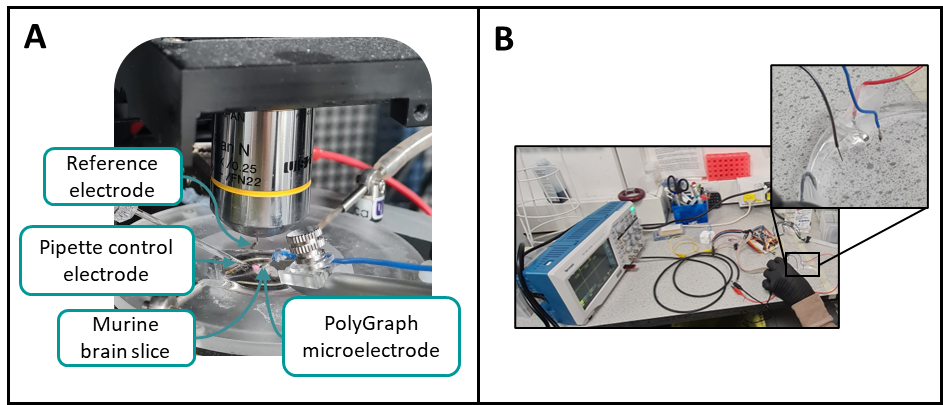
*

***Figure S17. Electrophysiological recording and stimulation with PolyGraph microneedles: A)*** *Experimental setup for recording local field potentials from murine brain slices, showing reference, control pipette, and PolyGraph microelectrodes.* ***B)*** *Stimulation setup with oscilloscope capturing waveform delivered through PolyGraph microneedle.*

*
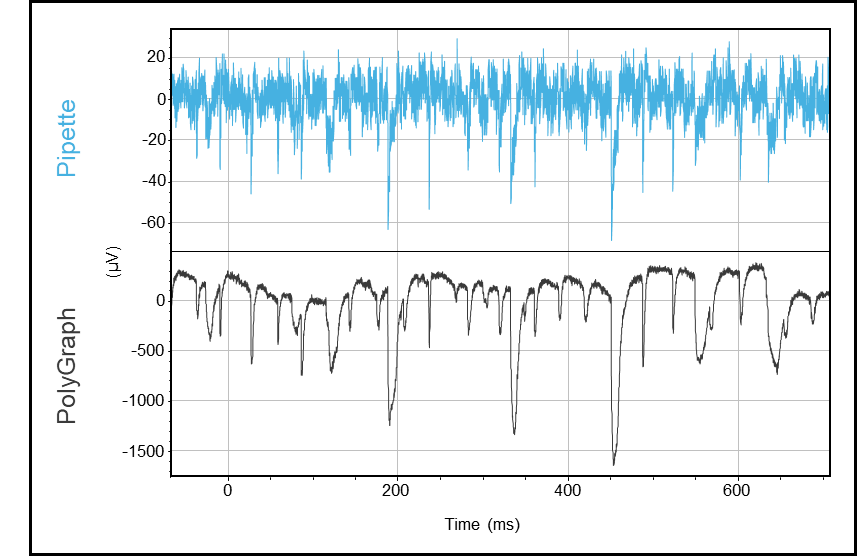
*

***Figure S18. Representative raw electrophysiological trace for PolyGraph & pipette electrodes:*** *Raw trace, showcasing increased signal-to-noise ratio (SNR) and high correlation of event waveforms recorded with PolyGraph electrodes. Y-scaling arbitrary due to differing amplifier gains.*

***
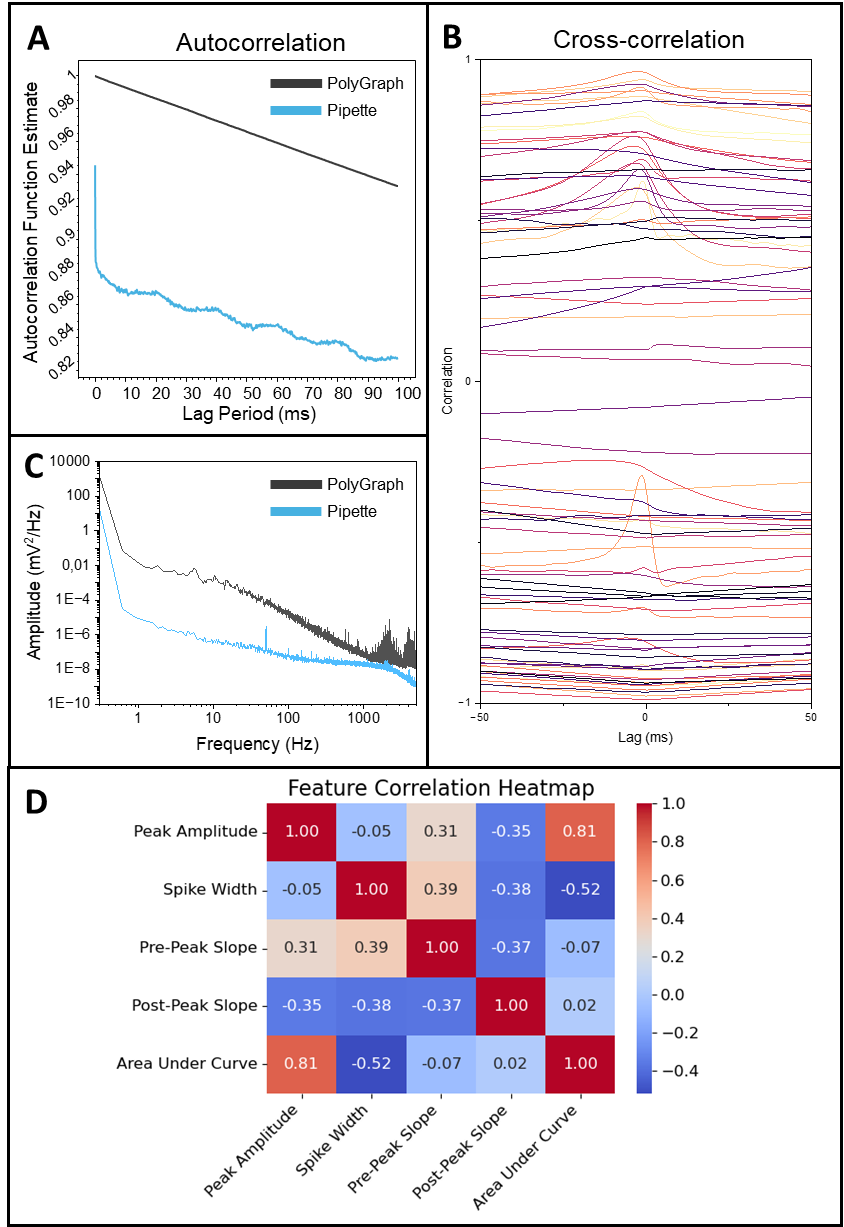
Figure S19. Correlation analysis of electrophysiological recordings: A)*** *Autocorrelation plot, indicating the temporal coherence of PolyGraph and pipette electrodes, and their relationship with themselves in the time domain, indicating lower noise and higher temporal stability in the PolyGraph signal.* ***B)*** *Cross-correlation plots of matching sweeps for the PolyGraph and pipette electrodes, with strong correlation corroborating physiological origin of recorded events.* ***C)*** *Power spectra for each electrode, indicating higher power at all frequencies for the PolyGraph electrode, with positive implications for signal-to-noise ratio.* ***D)*** *Heatmap of correlations between extracted event features.*

*
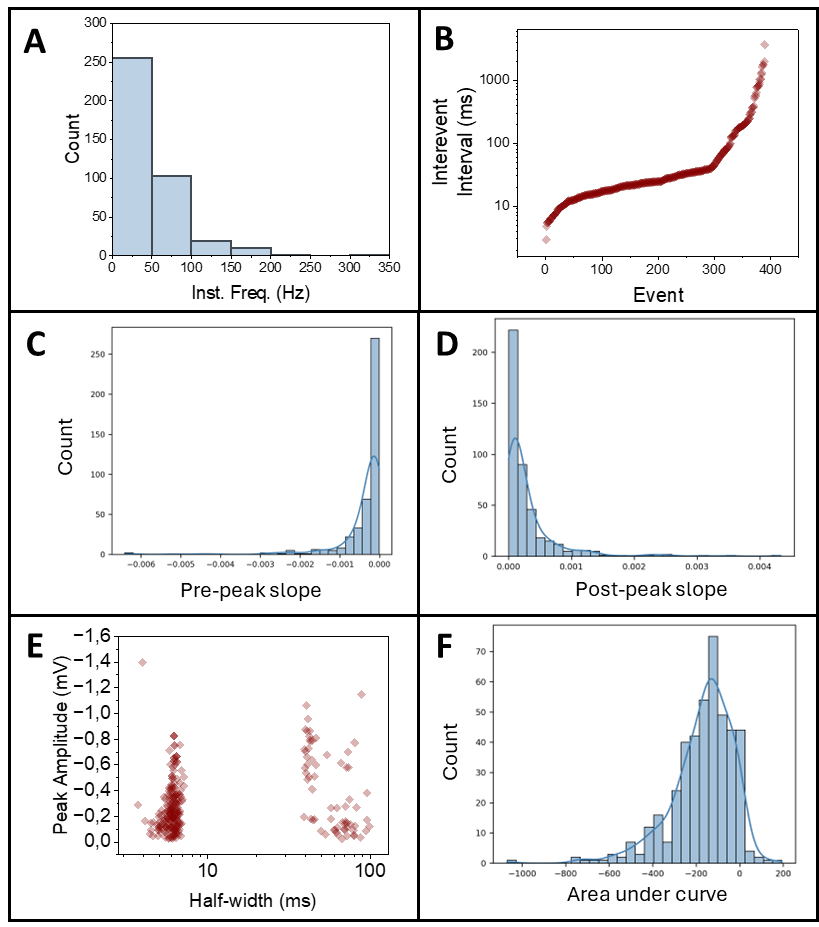
*

***Figure S20. Event statistics for recordings using PolyGraph electrode: A)****Instantaneous event frequency distribution.* ***B)*** *Interevent intervals, showing burst-like behaviour.* ***C‑D)****Histogram of pre- (C) and post-peak (D) slopes of events.* ***E)*** *Scatter plot of peak amplitude vs half-width of events.*  ***F)*** *Histogram of area under event trace, indicative of overall event magnitude.*

*
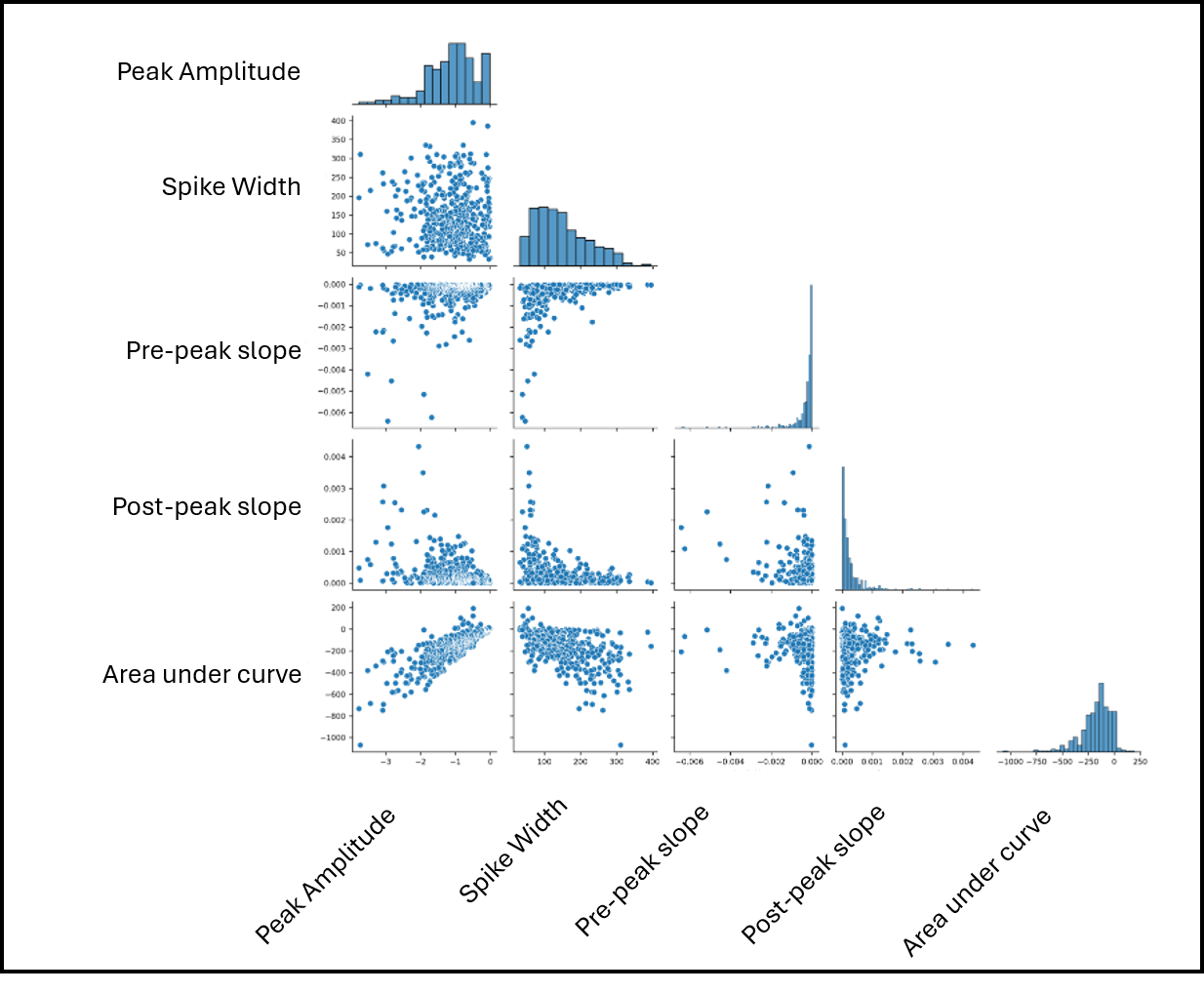
* ***Figure S21. Pairwise feature analysis of events recorded by PolyGraph electrodes:*** *Paired scatter plots and histograms of extracted event features (peak amplitude, spike width, pre- and post-peak slopes, and area under curve), showing relationships and distribution across the dataset.*

***
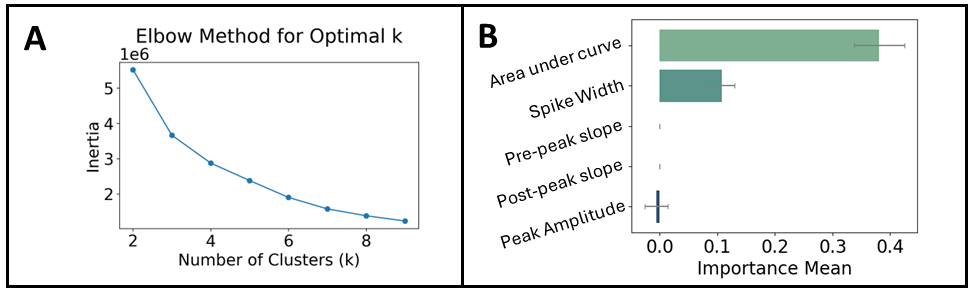
***

***Figure S22. Clustering and classification of PolyGraph-recorded events: A)*** *Elbow plot determining optimal number of clusters (k) for k-means clustering.* ***B)*** *Feature importance plot for MLP classifier, showing relative contribution of each feature to classification outcome.*

*
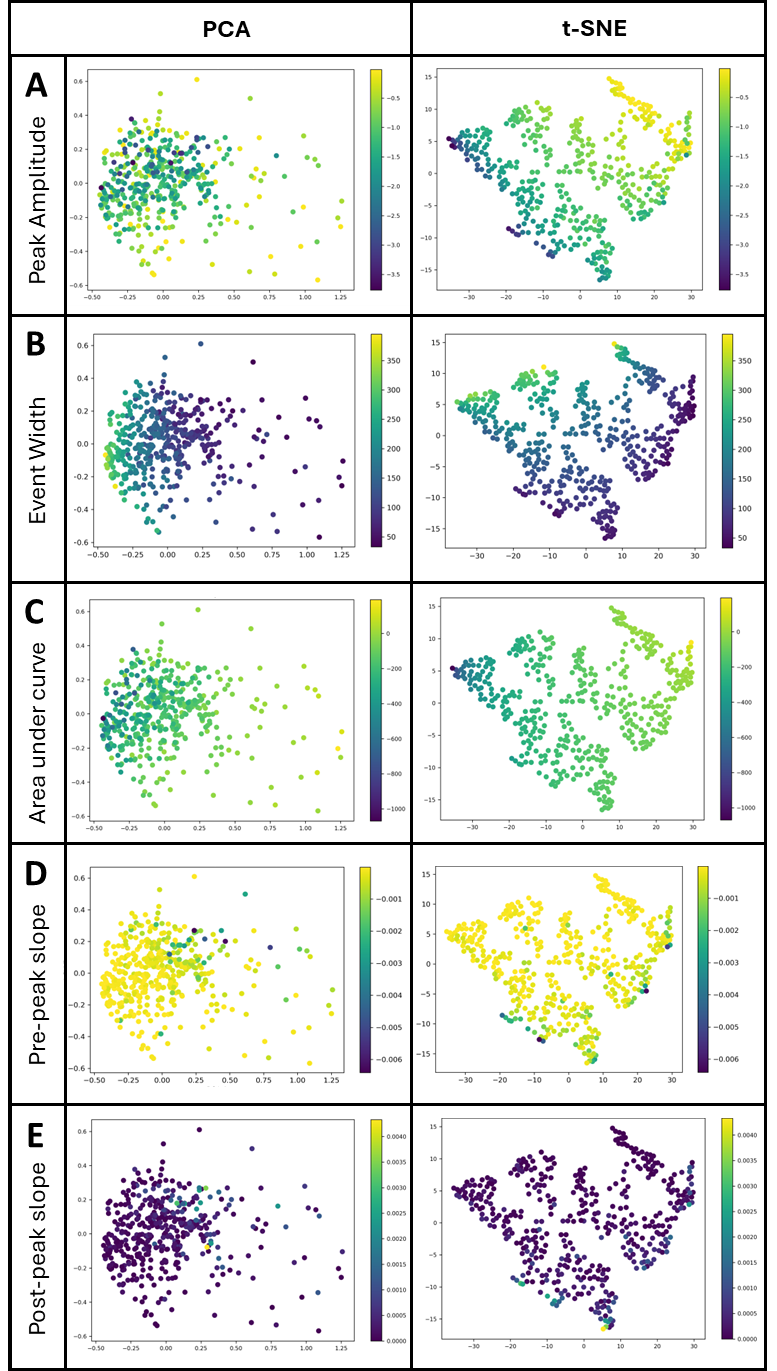
****Figure S23. Dimensionality reduction of event features for clustering: A-E)*** *Scatter plot results of principal component analysis (PCA, left) and t-distributed Stochastic Neighbour Embedding (t-SNE, right) methods for feature clustering, coloured according to peak amplitude (A), event width (B), area under curve (C), pre-peak slope (D), and post-peak slope (E).*
